# Network-driven analysis of human–*Plasmodium falciparum* interactome: processes for malaria drug discovery and extracting in silico targets

**DOI:** 10.1186/s12936-021-03955-0

**Published:** 2021-10-26

**Authors:** Francis E. Agamah, Delesa Damena, Michelle Skelton, Anita Ghansah, Gaston K. Mazandu, Emile R. Chimusa

**Affiliations:** 1 Division of Human Genetics, Department of Pathology, Institute of Infectious Disease and Molecular Medicine, Faculty of Health Sciences, University of Cape Town, Cape Town, South Africa; 2 Computational Biology Division, Department of Integrative Biomedical Sciences, Institute of Infectious Disease and Molecular Medicine, Faculty of Health Sciences, University of Cape Town, Cape Town, South Africa; 3 College of Health Sciences, Noguchi Memorial Institute for Medical Research, University of Ghana, P.O. Box LG 581, Legon, Ghana; 4 African Institute for Mathematical Sciences, 5-7 Melrose Road, Muizenberg, Cape Town, 7945 South Africa

**Keywords:** Malaria, Drug resistance, Genomics, Multi-omics, Gene ontology, Protein–protein interaction

## Abstract

**Background:**

The emergence and spread of malaria drug resistance have resulted in the need to understand disease mechanisms and importantly identify essential targets and potential drug candidates. Malaria infection involves the complex interaction between the host and pathogen, thus, functional interactions between human and *Plasmodium falciparum* is essential to obtain a holistic view of the genetic architecture of malaria. Several functional interaction studies have extended the understanding of malaria disease and integrating such datasets would provide further insights towards understanding drug resistance and/or genetic resistance/susceptibility, disease pathogenesis, and drug discovery.

**Methods:**

This study curated and analysed data including pathogen and host selective genes, host and pathogen protein sequence data, protein–protein interaction datasets, and drug data from literature and databases to perform human-host and *P. falciparum* network-based analysis. An integrative computational framework is presented that was developed and found to be reasonably accurate based on various evaluations, applications, and experimental evidence of outputs produced, from data-driven analysis.

**Results:**

This approach revealed 8 hub protein targets essential for parasite and human host-directed malaria drug therapy. In a semantic similarity approach, 26 potential repurposable drugs involved in regulating host immune response to inflammatory-driven disorders and/or inhibiting residual malaria infection that can be appropriated for malaria treatment. Further analysis of host–pathogen network shortest paths enabled the prediction of immune-related biological processes and pathways subverted by *P. falciparum* to increase its within-host survival.

**Conclusions:**

Host–pathogen network analysis reveals potential drug targets and biological processes and pathways subverted by *P. falciparum* to enhance its within malaria host survival. The results presented have implications for drug discovery and will inform experimental studies.

**Supplementary Information:**

The online version contains supplementary material available at 10.1186/s12936-021-03955-0.

## Background

*Plasmodium falciparum* malaria is a common infectious disease in Africa, and arguably the most important parasitic disease in the world, posing a significant public health burden as compared to other World Health Organization (WHO) disease-endemic regions. For instance, Africa contributed to about 93% (213 million of 228 million) and 94% (380,000 of 405,000) of global cases and deaths, respectively in 2018 [1].

The use of anti-malarial drugs has been the optimal avenue for controlling the disease. Currently, artemisinin-based combination therapy (ACT) is used as the first-line option for malaria treatment globally [2]. ACT was adopted in Africa after the decline in efficacy of previous widely used anti-malarial drugs, including chloroquine and sulfadoxine-pyrimethamine (SP) [2]. This was to ensure that, each component of the combinatorial drug acts through different mechanisms within the parasite, aiming to significantly reduce the likelihood of the emergence of multi-drug resistant parasites. Unfortunately, the parasite has shown tremendous ability to develop resistance and tolerance to these artemisinin derivatives and the long half-life partner drugs in some countries of the Greater Mekong Sub-region [2–4]. With several reports supporting parasite recrudescence and a significant decrease in their sensitivity to ACT, there has been continuous surveillance to monitor the emergence and spread of artemisinin-resistant parasite strains in Africa and elucidate whether it will follow a similar pattern observed for chloroquine and SP resistance where resistant strains originated from Southeast Asia [2, 4–7]. Interestingly, a study conducted by Uwimana et al. [7] has demonstrated the independent emergence and local spread of artemisinin partial resistance in Rwanda driven by R561H mutation in *kelch* gene. Another study conducted in Northern Uganda has also reported independent emergence and local spread of artemisinin-resistant parasite driven by mutations in the A675V or C469Y allele in the *kelch13* gene [8]. These pieces of evidence suggest that artemisinin resistance has emerged independently in Eastern Africa.

Researchers have proposed that the emergence of artemisinin parasite-resistant strains in Africa would result in about 78 million additional cases [4] and over 100,000 deaths annually [9]. Evidence abounds to the fact that a major challenge to controlling, eliminating, and eradicating malaria is drug resistance. It is the principal reason for the expansion of this life-threatening disease.

The architectural framework of the parasite’s genome constitutes a major framework influencing variations in the levels of the drug susceptibility, particularly having elucidated that *P. falciparum* anti-malarial drug resistance involves a single major gene effect. Spontaneous alterations in the form of single nucleotide variation and multiple mutations in different genes within the parasite genome capacitate the pathogen’s ability to develop tolerance mechanisms or resist the drug action over time thus, yielding the unexpected result. Genetic polymorphisms of known drug-resistance genes, such as *pfcrt*, *pfmdr1, pfk13, pfmrp1, pfdhfr,* and *pfdhps* generally express effects that counteract drugs controlling the disease [7, 10–12]. Compared to the clinical phenotype of resistance to quinolones and SP which usually takes the form of reduced accumulation of drugs within the parasite, particularly targets, artemisinin resistance, manifests as slow parasite clearance in patients and is characterized by the parasite’s ability to alter intraerythrocytic cell cycle with an increased ring stage and a shortened trophozoite stage [8, 13].

Falciparum malaria is a multifactorial disease that involves the complex interplay between the host, vector, and the pathogen [14, 15]. The host–pathogen interactions have been a driving selective force influencing the genetic architecture of both species, particularly, on how their genes are involved in drug and/or genetic resistance, disease susceptibility, and the infection processes [14, 16, 17].

Understanding these interactions requires an in-depth analysis of the organism’s proteome which is regarded to execute the genetic programme. Proteins execute functions mostly through extended networks with each other thereby forming a framework of the sensitive and complex regulatory system underlying a wide degree of post-translational modifications and processes [18]. The complex physicochemical dynamic connections formed within the system facilitate the structural and functional organization of the organism. These connections make up the protein–protein interaction network (PPIN).

Recent advances in host and parasite genomics in terms of high-throughput proteomics studies, host and parasite genome sequencing have led to a corresponding increase in biological datasets that describe the transition of species over time, particularly, the metabolic and developmental stages of pathogens. As such, the application of computational approaches to efficiently mine the inter and intra-species functional interactions to address the challenges presented by the disease is critical [19]. A systematic and comprehensive study of these complex interactions is essential in elucidating relevant pathways, signalling, drug resistance patterns, genes-gene products inter-relationships, and drug targets as well as developing novel hypotheses and models to predict disease causality [20].

In this study, a network-based integrative computational framework was leveraged to predict protein targets that may be used to guide the rational design of pathogen- and host-directed therapies for malaria treatment. Following the target prediction, a semantic similarity approach was implemented to prioritize informed potentially repurposable drugs that can be engineered for malaria treatment. Further analysis of host–pathogen network shortest paths enabled the prediction of immune-related biological processes and pathways potentially subverted by *P*. *falciparum* to increase its within-host survival.

## Methods

### Study design and procedures

**Fig. 1:**
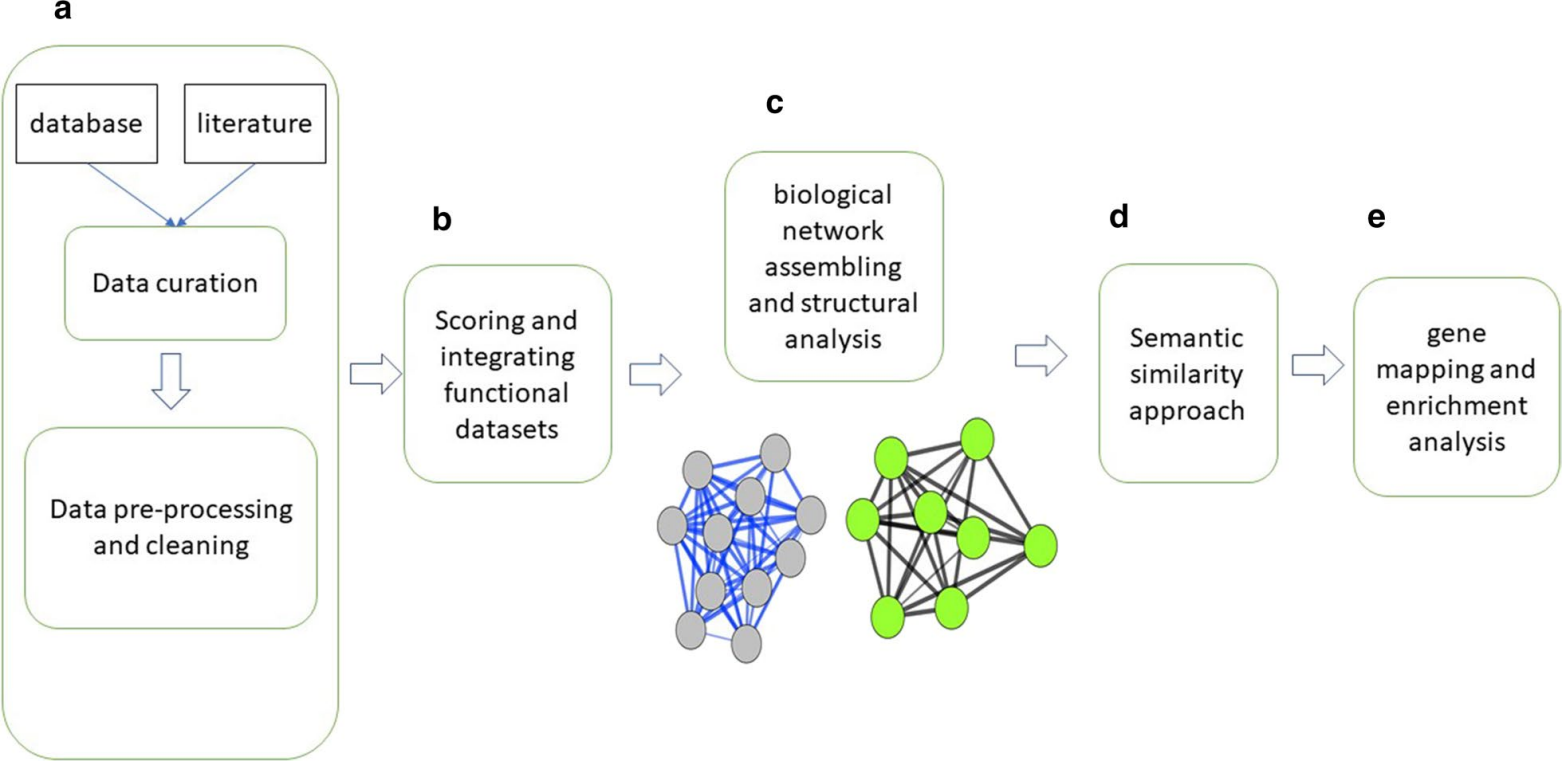
An overview of the approach implemented in this study

Various open access heterogeneous genomic and functional datasets retrieved from databases and literature using text mining techniques were used as inputs for analysis. The approach for this study (Fig. 1) consisted of five main steps: (1) data curation and pre-processing, (2) scoring and integrating functional datasets; (3) biological network assembling and structural analysis; (4) gene mapping and enrichment analysis (5) implicit semantic similarity approaches to predict malaria-similar diseases and repurposable drugs. Briefly, the framework uses integrative, scoring, and clustering algorithms coupled with statistical methods and biological knowledge to analyse and validate results.

### Data pre-processing

The various datasets utilized for this study are described in Additional file 4: Table S1. To achieve uniform identifiers (IDs) and convenient data manipulation, all genes and protein IDs were mapped to only reviewed proteins from Swiss-Prot under the non-redundant UniProt identifier system for harmonization. Human and *P. falciparum* genes were mapped to UniProt proteins with taxon identifier 9609 and 36,329 (*Plasmodium falciparum* 3D7 strain), respectively. Genes with no corresponding UniProt protein ID as at the time of this study were discarded.

Human malaria susceptibility-associated single nucleotide polymorphisms (SNPs) were retrieved from GWAS summary statistics datasets obtained from MalariaGEN [21]. The summary statistics dataset comprised of 20,273,529 spanning across chromosome one (1) to twenty-two (22). In this study, approximately 690,000 significant SNPs (p-value < 0.05) were filtered for further analysis. These SNPs were then mapped onto 44 genes (herein referred to as host candidate genes, Additional file 5: Table S2) using the dbSNP annotated data [22, 23].

### Scoring and integrating functional datasets

The study performed pathogen-pathogen, pathogen-host, and host-host protein sequence BLAST using their respective protein sequences retrieved from the UniProt database [24]. This was followed by implementing an information-theoretic based functional scoring scheme outlined by Mazandu and Mulder [25] and summarized in the Additional file 10: (Eqs. 1–8) to score the functional associations obtained from sequence BLAST and the conserved domains interaction datasets from the InterPro database [26].

### Scoring high-throughput experimental datasets and interologs

To incorporate curated functional interaction datasets in the analysis, the following criteria were defined to prioritize and score pair-wise interactions from experimental and interolog datasets retrieved from Reactome [27], IntAct [28], MINT [29], BIOGRID [30], and literature [31–36]. The criteria for scoring were based on; (1) the number of experimental methods that have confirmed such pair-wise functional interaction, (2) the number of databases that have reported such pair-wise functional interaction, and (3) the number of times the pair-wise functional interaction has been reported in the literature. For every pair-wise functional interaction supported by one evidence, a reliability score of 0.3 was assigned, else, a reliability score of 0.7 if it is supported by two or more pieces of evidence.

### Biological network assembling and structural analysis

Table 1 describes the number of proteins retrieved from each dataset, the number of reviewed proteins/genes considered from each input dataset and the pair-wise functional interaction implemented for further downstream analysis. From the pre-processed scored datasets, the functional interactions obtained were categorized, as low (scores less than 0.3), medium (scores ranging between 0.3 and 0.7), and high confidence levels (scores greater than 0.7). Biases may exist in the PPI network generated due to relatively high noise related to high-throughput data or experiments from which interactions are derived. In the absence of gold standard PPIs, integrating data from different sources and applying strict interaction reliability or confidence score cut-off are used to reduce the impact of these biases, leading to a PPI network of high confidence interactions with an increased coverage [37]. Further analyses only used medium and high confidence interactions or interactions predicted by two different sources. To evaluate the structural features of nodes (proteins) and edges (interactions), network centrality metrics including node degree, betweenness, and closeness (Additional file 10: Eqs. 9–11) were computed. High degree nodes with low betweenness describe degree-based or ‘local’ subnetwork interconnectivity mostly between functionally related proteins. High degree nodes with high betweenness contribute to structural-based or ‘global’ subnetwork interconnectivity and signal transmission thus, promoting system-level functional integration. Node closeness describes the average shortest length between neighbouring nodes determining the proximity to information sharing and biological process execution between functionally related nodes [38].

**Table 1:** Extracted functional interactions between manually annotated proteins

Interaction source	Number of Interactions	Number of proteins	Number of reviewed proteins	Number of pair-wise interactions between reviewed proteins	References
A. Functional interactions between annotated Plasmodium falciparum proteins					
LaCount P. falciparum PPI	2864	1308	62	17	[35]
Wuchty et al. in silico Plasmodium PPI (1)	19,979	2321	85	74	[31]
Wuchty et al. experimental PPI (2)	1428	361	12	11	[32]
Wuchty experimental P. falciparum PPI (3)	5458	1986	91	32	[33]
Wuchty et al. experimental P. falciparum PPI (4)	4918	1872	81	15	[34]
IntAct	2916	1343	67	26	[28]
InterPro	1013	256	98	241	[26]
Scored BLAST sequence similarity	1090 (BLAST)	163	130	231	[85]
STRING	617	163	114	386	[86]
B. Functional interactions between annotated human proteins					
Reactome	79,619	8059	5029	19,736	[27]
Score BLAST sequence similarity	3,807,888 (BLAST)	20,395	9611	143,533	[85]
InterPro	2,646,550	35,928	17,797	231,799	[26]
Bossi and Lerner	80,922	10,229	8416	54,238	[36]
STRING	11,759,454	19,354	18,836	5,244,655	[86]
IntAct	456,263	35,770	16,061	169,627	[28]

### Community structure and hub classification

The study aimed to identify hub genes/proteins that establish links with multiple functional clusters (communities), thus, characterized by both ‘local’ and ‘global’ network interconnectivity, structural, and functional features. To predict the hubs, clustering analysis was performed to identify network communities of densely connected nodes using a variant of an integrative computational algorithm that implements the Blondel et al. [39] heuristic method based on modularity optimization. This clustering model is a scalable hierarchical agglomerative method based on modularity optimization and has been shown to outperform all other known community detection methods [40], including Smart Local Moving [41], Infomap [42], and Label Propagation [43], in terms of computation time or complexity and the quality of the communities detected (modularity). The parasite candidate genes (herein referring to known antimalarial resistant genes and reported genes expressing signature of selection towards drug resistance) retrieved from literature [2, 6, 10] and host candidate gene-encoded proteins (Additional file 5: Table S2) were mapped onto the assembled parasite and host networks to cluster the networks. The subnetworks were explored to identify global hubs, herein defined as candidate gene/proteins characterized by a high degree and high betweenness score.

### Functional annotation analysis

Gene annotation and enrichment analysis were performed to elucidate statistically significant biological processes and pathways to which the hub genes are involved. Biological processes were inferred from the gene ontology database [44], whereas pathway information was obtained from PlasmoDB v46 [45] and the KEGG database [46]. By applying the hypergeometric test [47], p-values of processes and pathways were estimated, leveraging on their frequency of occurrence. The Bonferroni multiple correction test [47] was then implemented to estimate the adjusted p-values.

### Semantic similarity

The development of human disease ontology terms [48] has provided an enriched platform of human disease data to evaluate similarities between various diseases of different disorder classes based on gene-related molecular functions. The analysis is based on the hypothesis that varying combinations of disease-associated genes can influence the pathogenicity of similar diseases [49]. To predict repurposable drugs for malaria treatment, an in-house python-based semantic model was implemented for disease and drug similarity. The model uses host candidate key proteins, disease-target datasets, and gene ontology datasets as input data to make predictions based on functional similarities inferred from associated gene ontology terms. The semantic similarity approach was further implemented to identify diseases that are biologically similar to malaria. In the analysis, the semantic similarity score between the pair of diseases was leveraged to identify and prioritize diseases similar to malaria. The similarity score was estimated by computing the Kappa statistic, Jaccard, and the Best Match Average (BMA) measures (Additional file 10). The score is a quantitative measure of the underlying shared biological processes among the disease targets. A higher score between disease enriched processes suggests that the disease-pair and their associated candidate proteins are functionally similar thus, the likelihood for similar treatment options. A similarity score threshold was defined based on the upper quartile and interquartile range of the distribution given by $tr = Q3+\varepsilon *IQR$, where $\varepsilon$, $tr, Q3$ and $IQR$ represent the tuning parameter $(0\le \varepsilon \le 1.5)$ threshold, upper quartile, and interquartile range, respectively.

## Results

### Network clustering and functional annotation analysis

**Fig. 2:**
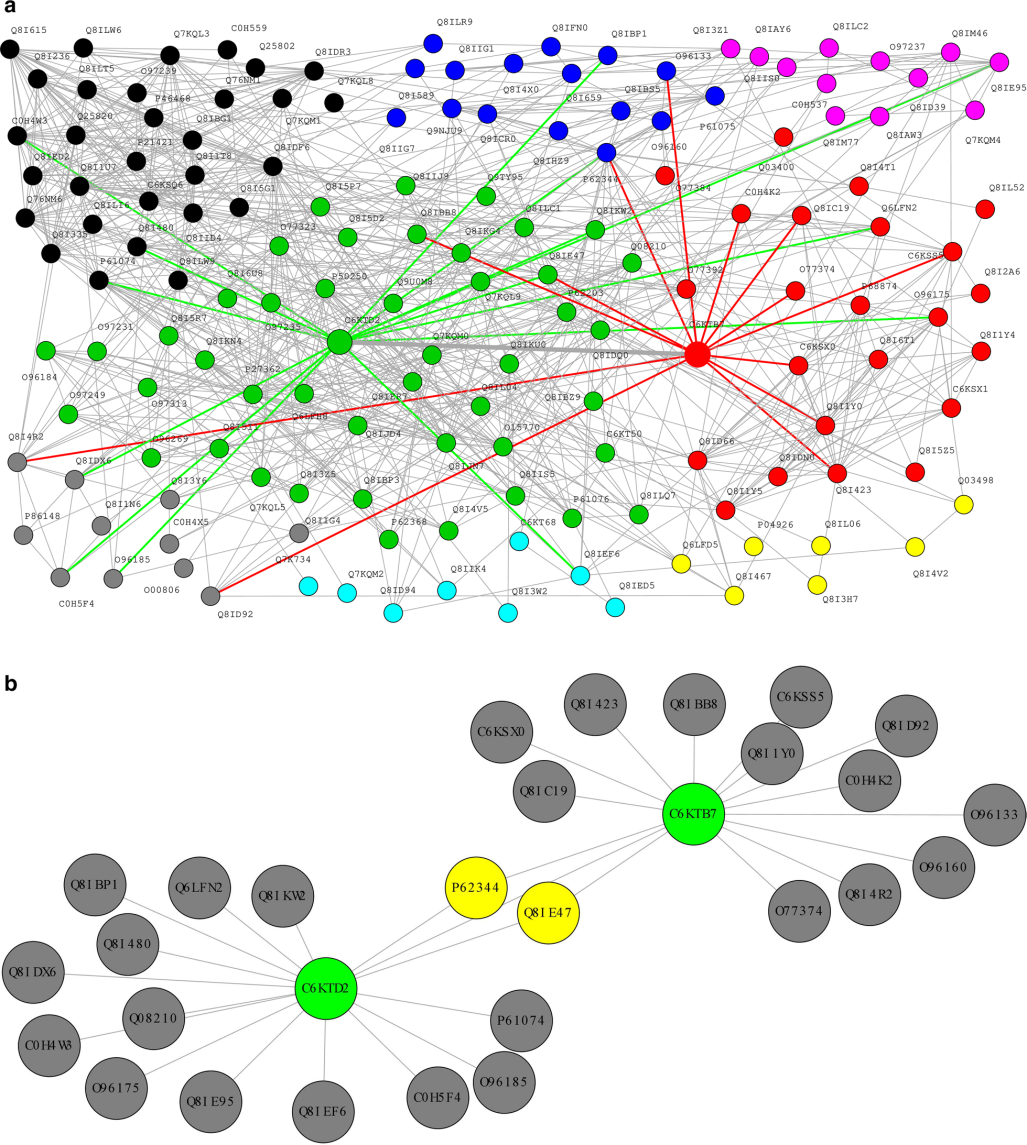
**A** Assembled parasite network and **B** Functional interactions between ***C6KTD2*** and ***C6KTB7*** subnetwork within the parasite network. The nodes common to the subnetworks are coloured in yellow

The generated parasite network consists of 662 unique interactions among 140 characterized proteins (Fig. 2A). The unified host network assembled comprised of 4,133,136 unique functional interactions between 20,329 nodes. The host-parasite network consisted of 31,512 unique functional interactions between 8023 proteins. The topology properties of the generated networks were explored to investigate the relationships between the degree, betweenness, and closeness centrality measures. As shown in Additional file 1: Fig. S1, subnetworks were classified as either degree-based (subnetworks formed from nodes with a high degree but low betweenness) or structural-based (subnetworks formed from nodes with high degree, high betweenness, and high closeness). The nodes forming the degree-based and structural-based subnetworks are herein referred to as key proteins.

### Network clustering analysis reveals disease candidate key proteins/genes as hubs

The purpose of clustering is to partition the complex network into subnetworks and identify essential communities and critical functional nodes. It is a way of grouping nodes in the network into modules sharing functional connectivity. The parasite network (Fig. 2A) consists of 8 clusters of which 5 contained key proteins whereas the dense human network consisted of 32 clusters of which 7 contained key proteins. From the network clustering (Additional file 2: Fig. S2A, Additional file 3: Fig. S2B), two parasite candidate key proteins were identified as hubs, C6KTD2 *(SET1)* and C6KTB7 *(PFF1365c)* both on chromosome 6. These parasite candidate key proteins are involved in the merozoite developmental stage where they invade red blood cells (RBCs), cause disease severity, and contribute to the exponential growth of the parasite population [50]. Analysis of the host network revealed 6 candidate key proteins as hubs; P22301 *(IL10 [MIM: 124092]),* P05362 *(ICAM1 [MIM: 147840]),* P01375 *(TNF [MIM: 191160]),* P30480 *(HLA-B [MIM: 142830]),* P16284 *(PECAM1 [MIM: 173445])* and O00206 *(TLR4 [MIM: 603030])*. These proteins are cognate host receptors that respond to inflammation by releasing pro-inflammatory cytokines, enhancing adhesion of parasitized red blood cells (RBCs), parasite sequestration in organs rupture, and removal of infected RBCs [50, 51]. Most importantly, the identified host candidate key proteins are targets for drugs in DrugBank [52] and have been reported to offer higher opportunities for drug repurposing, although a smaller proportion of the human genome is druggable [53–55]. Additional file 6: Table S3 and Additional file 7: Table S4 describe the identified candidate key proteins prioritized by the degree, betweenness, and closeness scores.

### Biological processes and pathway enrichment of hub genes

The identified hub genes within the subnetworks were used for the functional annotation process. The results revealed 4 statistically significant essential processes and an enriched pathway (Table 2) specific to the parasite key hub genes. A total of 23 significant biological processes and 21 enriched pathways (Table 3) were identified to underly host hub gene's contribution towards malaria infection. From the host perspective, the hub genes are mainly involved in immune regulatory biological processes within immune-related pathways (47.6%), parasitic disease-related pathways (23.8%), bacteria disease-related pathways (14.2%), endocrine and metabolic disease-related pathways (4.7%), viral disease-related pathway (4.7%) and transport and catabolism related pathway (4.7%)[44, 46]. Most importantly, the malaria pathway ranked the most significant pathway with both p-value and adjusted p-value of 0. This supports the association of these hub genes to malaria. The enriched pathways presented the likelihood of similarity between malaria and other diseases.

**Table 2:** Statistically significant biological processes and pathways of key *P. falciparum* malaria-associated genes inferred from PlasmoDB v46 and gene ontology database

Enriched biological process			
Gene Ontology (GO)-ID Process	Gene ontology term name	P-value	Adjusted p-value
GO:0019904	Protein domain specific binding	1.77e−3	7.08e-3
GO:0004842	Ubiquitin-protein transferase activity	5.31e−3	4.25e-2
GO:0019787	Ubiquitin-like protein transferase activity	5.75e−3	4.60e-2
GO:0051568	Histone h3-k4 methylation	0.0103149	0.030945
A. Enriched pathway			
Pathway ID	Pathway-name	P-value	Adjusted p-value
ec00310	Lysine degradation	3.87e-2	3.87e−2

**Table 3:** Statistically significant biological processes and enriched pathways of key human malaria-associated genes inferred from gene ontology and KEGG database

Gene ontology (GO)-id process	Gene ontology term name	P-value	Adjusted P-value
Enriched biological process			
Go:0042346	Positive regulation of nfkappab import into nucleus	2.00161e−05	0.00432
Go:0045348	Positive regulation of mhc Class ii biosynthetic process	2.40637e−06	0.00052
Go:0032689	Negative regulation of Interferon-gamma production	3.10034e−05	0.00670
Go:0007157	Heterophilic cell–cell adhesion Via plasma membrane cell adhesion molecules	0.00012	0.02714
Go:2000352	Negative regulation of endothelial Cell apoptotic process	2.70760e−05	0.00585
Go:0032715	Negative regulation of interleukin-6 production	8.99764e−08	1.9434e-05
Go:2000343	Positive regulation of chemokine (c-x-c motif) ligand	1.68841e−05	0.00364
Go:0032729	Positive regulation of Interferon-gamma production	0.00012	0.02713
Go:0070374	Positive regulation of erk1 And erk2 cascade	2.8883e−05	0.00623
Go:0050830	Defence response to gram-positive bacterium	2.65221e−05	0.00572
Go:0034116	Positive regulation of heterotypic cell–cell adhesion	1.68841e−05	0.00364
Go:0044130	Negative regulation of Growth of symbiont in host	6.07930e−06	0.00131
Go:0030198	Extracellular matrix organization	5.39819e−05	0.01166
Go:0045416	Positive regulation of Interleukin-8 biosynthetic process	2.40637e−06	0.00051
Go:0032755	Positive regulation of Interleukin-6 production	0.00016	0.03562
Go:0002740	Negative regulation of cytokine Secretion involved in immune response	1.00303e−06	0.00021
Go:0045429	Positive regulation of nitric Oxide biosynthetic process	1.23374e−07	2.665e−05
Go:0043032	Positive regulation of Macrophage activation	1.02165e−05	0.00220
Go:1904999	Positive regulation of leukocyte Adhesion to arterial endothelial Cell	2.00663e−07	4.3343e−05
Go:0031663	Lipopolysaccharide mediated Signalling pathway	2.42641e−08	5.2410e−06
Go:1904707	Positive regulation of vascular Smooth muscle cell proliferation	0.00016	0.03663
Go:0032800	Receptor biosynthetic process	6.68766e−07	0.00014
Go:1900227	Positive regulation of nlrp3 inflammasome complex assembly	2.70759e−05	0.00584
A. Enriched pathway			
Kegg pathway id	Kegg-pathway-name	P-value	Adjusted p-value
Hsa05144	Malaria	0.0	0.0
Hsa05310	Asthma	6.52960e−07	7.966e−05
Hsa04145	Phagosome	1.46003e−06	0.00017
Hsa05146	Amoebiasis	0.00014	0.01745
Hsa04640	Hematopoietic cell lineage	1.70661e−06	0.00020
Hsa05330	Allograft rejection	3.03329e−06	0.00037
Hsa05133	Pertussis	8.77858e−06	0.00107
Hsa04940	Type i diabetes mellitus	9.00124e−05	0.01098
Hsa05162	Measles	8.93218e−05	0.0108
Hsa04650	Natural killer cell mediated cytotoxicity	0.00014	0.01724
Hsa04657	Il—17 signalling pathway	0.00037	0.04624
Hsa05152	Tuberculosis	1.92895e−10	2.35332e−08
Hsa05150	Staphylococcus aureus infection	4.40440e−08	5.37336e−06
Hsa05142	Chagas disease (american trypanosomiasis)	7.77032e−05	0.00947
Hsa05143	African trypanosomiasis	7.32790e−10	8.9400e-08
Hsa05140	Leishmaniasis	1.16192e−11	1.41754e−09
Hsa05321	Inflammatory bowel disease (ibd)	9.68744e−08	1.18186e−05
Hsa05322	Systemic lupus erythematosus	3.51918e−05	0.00429
Hsa05323	Rheumatoid arthritis	0.00027	0.03331
Hsa05320	Autoimmune thyroid disease	9.62632e−06	0.00117
Hsa05332	Graft—versus—host disease	0.00010	0.01229

### Shortest path analysis between hub genes reveals functional insights towards disease progression

The study investigated functional interactions between the host and pathogen targets in the context of parasite survival, host immune tolerance, and how it can inform drug discovery research. The immune tolerance machinery remains to be the natural driving force influencing the parasite's survival when host–pathogen recognition receptors sense infection. To contribute to this effort, the shortest paths between the parasite and host hub proteins within the host-parasite network were explored to gain insight into the most likely routes for innate immune response interference by the parasite.

Studies have shown that the shortest path analysis of a functional network yields high coverage compared to direct neighbours within the network [56]. The shortest path between host–pathogen disease-associated candidate key genes herein refer to the minimum number of edges required to connect these genes. Longer paths consist of more nodes (proteins) involved in a cascade of signalling processes to trigger innate immune responses by inducing the production of chemokines and cytokines upon parasite infection. It is, therefore, a measure of information relay between the hub genes thus, the shorter the path, the quicker the transmission and the relevance of the interaction in investigating immune adaptiveness and parasite pathogenesis [56]. It is noteworthy that, shortest path lengths between the pathogen disease-associated genes and human disease-associated genes conferring immunity in the functional network are the most feasible routes of parasite invasion of host immunity and escaping the contribution of host genetics towards drug action [56, 57]. Most importantly, shortest paths would trigger excessive activation which may be deleterious as it can cause systemic inflammation and disease [50]. This, therefore, suggests that developing immune-modulatory drugs that target the host targets can induce an immune response to avoid the state of been overwhelmed by the parasite.

The results showed that the shortest path between parasite hub proteins and any of the host hub proteins were between O00206—*C6KTB7,* and O00206-*C6KTD2* as shown in Table 4. Such paths were characterized by mediators. These mediators are mostly signal receptors involved in cell regulatory activities, production of cytokines, transcription processes, and regulating cell survival and apoptosis. The shortest paths identified (Table 4) suggest that inhibition or alteration to the proper functioning of each path might help the parasite to survive immune responses, thus, the aggregation of small effects. The development of adaptive immunity is expected to happen when the parasite undergoes diversity throughout time such that they evade the host system when they become tolerant and establish different mechanisms to interfere with the host’s response [58]. These interferences can also be in the form of the production of effector mechanisms that can down-regulate innate immunity [59]. The results have shown that the dynamic patterns to parasite survival and immune adaptiveness are mediated by other human-specific genes or proteins conferring immunity.

**Table 4:** Shortest paths linking O00206 (*TLR4*) and parasite hub nodes within the host–pathogen unified functional network

Host candidate key protein	Mediator	Mediator gene name [OMIM ID]	Mediator description	Parasite candidate key protein	Potential parasite adaptive biological process
A. Shortest paths linking O00206 and C6KTB7 (*SET1*) nodes within the host–pathogen unified functional network					
O00206	Q9BYH8	*NFKBIZ* [MIM: 608004]	NF-kappa-B inhibitor zeta	***C6KTB7***	Inflammatory response; T cell receptor signalling pathway
O00206	Q05823	*RNASEL* [MIM: 180435]	2-5A-dependent ribonuclease	***C6KTB7***	Interferon alpha/beta signalling, positive regulation of transcription by RNA polymerase II
O00206	Q5S007	*LRRK2* [MIM: 609007]	Leucine-rich repeat serine/threonine-protein kinase 2	***C6KTB7***	Activation of MAPK activity;
O00206	Q38SD2	LRRK1 [MIM: 610986]	Leucine-rich repeat serine/threonine-protein kinase 1	***C6KTB7***	Positive regulation of intracellular signal transduction
O00206	O75762	*TRPA1* [MIM: 604775]	Transient receptor potential cation channel subfamily A member 1	***C6KTB7***	Cell surface receptor signalling pathway
O00206	Q96HA7	*TONSL* [MIM: 604546]	Tonsoku-like protein	***C6KTB7***	Cytoplasmic sequestering of transcription factor
O00206	Q00653	*NFKB2* [MIM: 164012]	Nuclear factor NF-kappa-B p100 subunit	***C6KTB7***	Inflammatory response; innate immune response; NIK/NF-kappaB signalling; negative regulation of transcription by RNA polymerase II
O00206	P25963	*NFKBIA* [MIM: 164008]	NF-kappa-B inhibitor alpha	***C6KTB7***	Positive regulation of inflammatory response; apoptotic process; I-kappaB kinase/NF-kappaB signalling
O00206	P46531	*NOTCH1* [MIM: 190198]	Neurogenic locus notch homolog protein 1	***C6KTB7***	Immune response
O00206	P20749	*BCL3* [MIM: 109560]	B-cell lymphoma 3 protein	***C6KTB7***	Regulation of apoptotic process; regulation of interferon-gamma production; T-helper 1 type immune response; positive regulation of interferon-gamma production
O00206	P42771	*CDKN2A* [MIM: 600160]	Cyclin-dependent kinase inhibitor 2A	***C6KTB7***	Apoptotic process;
O00206	Q8NI38	*NFKBID* [MIM: 618887]	NF-kappa-B inhibitor delta	***C6KTB7***	Inflammatory response; innate immune response;
O00206	P19838	*NFKB1* [MIM: 164011]	Nuclear factor NF-kappa-B p105 subunit	***C6KTB7***	Apoptotic process; inflammatory response; innate immune response; regulation of transcription by RNA polymerase II
O00206	Q8NDB2	*BANK1* [MIM: 610292]	B-cell scaffold protein with ankyrin repeats	***C6KTB7***	B cell activation; positive regulation of interleukin-6 production
O00206	Q15653	*NFKBIB* [MIM: 604495]	NF-kappa-B inhibitor beta	***C6KTB7***	Signal transduction
B. Shortest paths linking O00206 and C6KTD2 within the host–pathogen unified functional network					
O00206	Q13114	*TRAF3* [MIM: 601896]	TNF receptor-associated factor 3	***C6KTD2***	Apoptotic process; innate immune response; toll-like receptor signalling pathway; regulation of interferon-beta production; positive regulation of JNK cascade;
O00206	Q86WT6	*TRIM69* [MIM: 616017]	E3 ubiquitin-protein ligase TRIM69	***C6KTD2***	Apoptotic process
O00206	Q12933	*TRAF2* [MIM: 601895]	TNF receptor-associated factor 2	***C6KTD2***	Positive regulation of JNK cascade; apoptotic process;
O00206	Q9UPN9	*TRIM33* [MIM: 605769]	E3 ubiquitin-protein ligase TRIM33	***C6KTD2***	Negative regulation of transcription by RNA polymerase II;
O00206	P55895	*RAG2* [MIM: 179616]	V(D)J recombination-activating protein 2	***C6KTD2***	B cell homeostatic proliferation;
O00206	O75626	*PRDM1* [MIM: 603423]	PR domain zinc finger protein 1	***C6KTD2***	Adaptive immune response; innate immune response; negative regulation of transcription by RNA polymerase II;
O00206	Q96CA5	*BIRC7* [MIM: 605737]	Baculoviral IAP repeat-containing protein 7	***C6KTD2***	Apoptotic process
O00206	Q8NHM5	*KDM2B* [MIM: 609078]	Lysine-specific demethylase 2B	***C6KTD2***	Negative regulation of transcription by RNA polymerase II; apoptotic process;
O00206	Q9BUZ4	*TRAF4* [MIM: 602464]	TNF receptor-associated factor 4	***C6KTD2***	Apoptotic process; regulation of I-kappaB kinase/NF-kappaB signalling;
O00206	Q96P53	*WDFY2* [MIM: 610418]	WD repeat and FYVE domain-containing protein 2	***C6KTD2***	Positive regulation of protein phosphorylation
O00206	P15918	*RAG1* [MIM: 179615]	V(D)J recombination-activating protein 1	***C6KTD2***	adaptive immune response; immune response;
O00206	P19474	*TRIM21* [MIM: 109092]	E3 ubiquitin-protein ligase TRIM21	***C6KTD2***	Innate immune response; response to interferon-gamma
O00206	Q9Y4K3	*TRAF6* [MIM: 602355]	TNF receptor-associated factor 6	***C6KTD2***	Activation of MAPK activity; positive regulation of JNK cascade; apoptotic process; toll-like receptor signalling pathway; regulation of transcription by RNA polymerase II
O00206	Q6PCT2	*FBXL19* [MIM: 609085]	F-box/LRR-repeat protein 19	***C6KTD2***	Post-translational protein modification
O00206	Q14258	*TRIM25* [MIM: 600453]	E3 ubiquitin/ISG15 ligase TRIM25	***C6KTD2***	Innate immune response;
O00206	Q9UNE7	*STUB1* [MIM: 607207]	E3 ubiquitin-protein ligase CHIP	***C6KTD2***	Protein ubiquitination
O00206	Q15075	*EEA1* [MIM: 605070]	Early endosome antigen 1	***C6KTD2***	Endocytosis
O00206	Q8IWB7	*WDFY1* [MIM: 618080]	WD repeat and FYVE domain-containing protein 1	***C6KTD2***	Positive regulation of toll-like receptor 3 and 4 signalling pathway
O00206	Q09472	*EP300* [MIM: 602700]	Histone acetyltransferase p300	***C6KTD2***	Apoptotic process; positive regulation of NIK/NF-kappaB signalling
O00206	Q8IYM9	*TRIM22* [MIM: 606559]	E3 ubiquitin-protein ligase TRIM22	***C6KTD2***	positive regulation of I-kappaB kinase/NF-kappaB signalling
O00206	Q9Y2K7	*KDM2A* [MIM: 605657]	Lysine-specific demethylase 2A	***C6KTD2***	Histone H3-K36 demethylation
O00206	Q9NQV6	*PRDM10* [MIM: 618319]	PR domain zinc finger protein 10	***C6KTD2***	Positive regulation of transcription by RNA polymerase II
O00206	Q92793	*CREBBP* [MIM: 600140]	CREB-binding protein	***C6KTD2***	Positive regulation of transcription by RNA polymerase II; apoptotic process
O00206	Q6UWE0	*LRSAM1* [MIM: 610933]	E3 ubiquitin-protein ligase LRSAM1	***C6KTD2***	Ubiquitin-dependent endocytosis;
O00206	Q8WVD3	*RNF138* [MIM: 616319]	E3 ubiquitin-protein ligase RNF138	***C6KTD2***	Protein ubiquitination
O00206	Q8TCQ1	*MARCHF1* [MIM: 613331]	E3 ubiquitin-protein ligase MARCHF1	***C6KTD2***	Immune response
O00206	Q6Q0C0	*TRAF7* [MIM: 606692]	E3 ubiquitin-protein ligase TRAF7	***C6KTD2***	Apoptotic process; positive regulation of MAPK cascade
O00206	O00463	*TRAF5* [MIM: 602356]	TNF receptor-associated factor 5	***C6KTD2***	Apoptotic process; positive regulation of I-kappaB kinase/NF-kappaB signalling; positive regulation of JNK cascade;
O00206	Q13233	*MAP3K1* [MIM: 600982]	Mitogen-activated protein kinase kinase kinase 1	***C6KTD2***	Activation of protein kinase activity
O00206	P98170	*XIAP* [MIM: 300079]	E3 ubiquitin-protein ligase XIAP	***C6KTD2***	Regulation of innate immune response; regulation of inflammatory response; apoptic process
O00206	Q6ZMZ0	*RNF19B* [MIM: 610872]	E3 ubiquitin-protein ligase RNF19B	***C6KTD2***	Adaptive immune response
O00206	O14964	*HGS* [MIM: 604375]	Hepatocyte growth factor-regulated tyrosine kinase substrate	***C6KTD2***	Regulation of MAP kinase activity; positive regulation of gene expression
O00206	Q9NWF9	*RNF216* [MIM:]	E3 ubiquitin-protein ligase RNF216	***C6KTD2***	Apoptotic process

Importantly, *pfk13* is known to be associated with artemisinin resistance, but little is known of its interaction with host genes/proteins and how that influences drug resistance or parasite survival within the host. Further network analysis was performed to explore interactions between *pfk13* and the host candidate key proteins. The results revealed no functional interactions between *pfk13* and the host hub genes. However, the analysis showed interactions between *pfk13* and highly expressed host kelch-like proteins and regulatory genes involved in essential processes such as transcription regulation, cell-surface, cell–cell signalling, and regulation of phosphorylation. Among the regulatory genes include the transcriptional regulator Kaiso (*ZBTB33*), Zinc finger and BTB domain-containing protein 17 (*ZBTB17 [MIM: 604084]*), BTB/POZ domain-containing protein 10 (*KCTD10 [MIM: 613421]*), Zinc finger and BTB domain-containing protein 10 (*ZBTB10 [MIM: 618576]*), Myoneurin (*MYNN [MIM: 606042]*), Nucleoprotein TPR (*TPR [MIM: 189940]*) and Gigaxonin (*GAN [MIM: 605379]*).

### Predicting repurposable drugs for malaria treatment based on Implicit Semantic Similarity

**Fig. 3:**
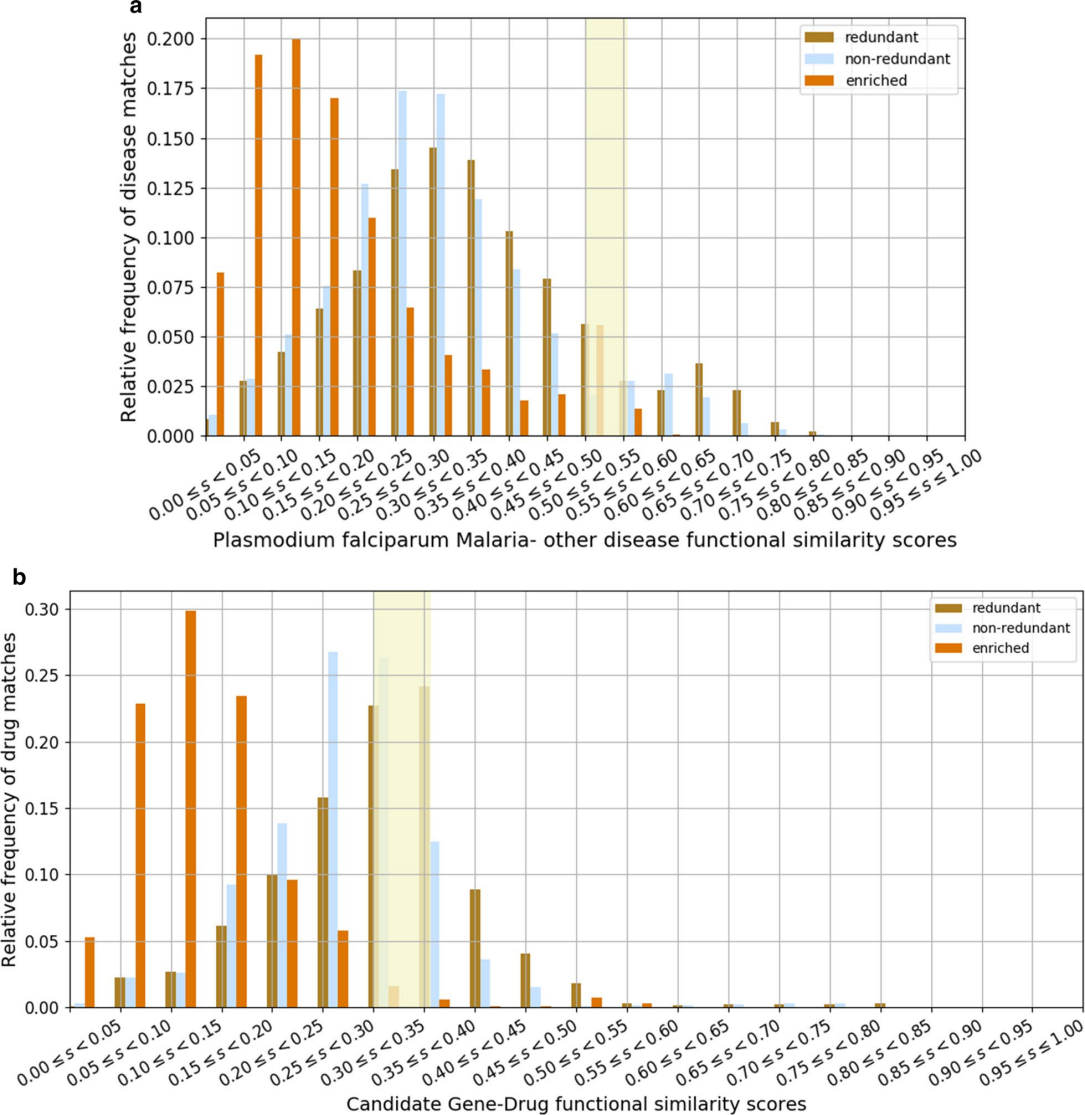
**A** Different distributions of disease similarity scores obtained in terms of frequencies (proportions) of disease matches vs similarity scores between disease-associated processes. The bigger rectangular bar indicates the threshold for the similarity between disease pairs of which the enriched similarity score (ESS) were used for further analysis. **B** Distributions of drug similarity scores obtained in terms of the relative frequency of drug matches against functional similarity scores between candidate gene and drug. The bigger rectangular bar indicates the threshold for the similarity between drug pairs of which the enriched similarity score (ESS) were used for further analysis

After defining a semantic similarity score threshold (as illustrated in Fig. 3A), 1944 (8.04%) out of 24,166 diseases in the DisGeNet platform version 6 were identified to be semantically like malaria. The disease hits were filtered by maintaining those whose targets are involved in the same pathways of host Malaria hub genes. The disease hits were further filtered by maintaining diseases supported by biological evidence from the literature. The final filtered disease hits consisted of 113 diseases (Additional file 8: Table S5). These identified diseases fall in the category of infectious, inflammatory, and genetic neurological diseases which trigger the human immune machinery to overproduce cytokines; confirming the fact that malaria is an inflammatory response-driven disease. Among the top disease hits includes sickle cell anaemia [MIM: 603903], liver dysfunction [MIM: 613759], fever ([MIM: 142680], [MIM: 614371]), hepatitis ([MIM: 606518], [MIM: 609532]) and respiratory distress syndrome [MIM: 267450]. It is interesting to note that the disease hits described have been reported to be governed by the same pathologic principles as malaria infection [60, 61].Finally, to predict repurposable drugs, 1426 approved drugs and their corresponding targets were retrieved from the DrugBank database. Next, non-human drugs were excluded and were remained with 1282 drugs and their targets for further downstream analysis. The drugs were further filtered to retain those with target processes associated with malaria and the predicted malaria similar diseases. Then after, the semantic approach was implemented to predict putative repurposable drugs. From the identified drugs sharing some similarities in terms of processes, those that are over 1.5 of the interquartile range were extracted and ordered. With a defined similarity score threshold of 0.31099875 (Fig. 3B) based on similarity in terms of processes the drugs are involved in, the results revealed 26 potential repurposable drugs (Additional file 9: Table S6).The repurposable drugs categorized as known anti-malarial, monoclonal antibodies, immunomodulators, herbs, natural products, Janus kinase inhibitors, and thrombolytic agents act as either antagonist, agonists, inhibitors, or precursors targeting genes over-represented in immune response and cytokine-mediated signalling processes. Janus kinase inhibitors including ruxolitinib, are known for their ability to effectively inhibit the production of cytokines and cause eryptosis contributing to the clearance of erythrocytes infected with malaria, decreased parasitaemia, and protection against severe malaria [62]. The results showed that drugs involved in regulating host immune response to inflammatory-driven disorders target the Tumour necrosis factor and inhibit its activity to regulate downstream processes such as pro-inflammatory cascade signalling. Several of the potentially repurposable drugs are used for treating some diseases like malaria including rheumatoid arthritis, ischemic stroke, psoriatic arthritis, and idiopathic arthritis.

The drug hits include chloroquine, infliximab, hydroxychloroquine, glucosamine, ginseng, minocycline, ruxolitinib, and natalizumab which can be appropriated for malaria treatment. These drug hits have been reported to control malaria infection by inhibiting residual malaria infection, knocking parasite gene expression, and activating eryptosis. Furthermore, some of the hits such as adalimumab, Natalizumab, etanercept, thalidomide, ustekinumab, and canakinumab are anti-TNF monoclonal antibodies and anti-inflammatory agents that could modulate the immune response to severe and cerebral malaria. The analysis also predicted thrombolytic agents such as anistreplase, reteplase, alteplase, and tenecteplase which can play an essential role in the treatment of coagulopathy in malaria, particularly among severe and cerebral malaria infections [63]. Considering malaria as an inflammatory-response driven disease presenting with multiple manifestations, these putative drug hits can undergo both computational and experimental repositioning for adjunctive malaria therapy, particularly severe and cerebral malaria.

## Discussion

**Fig. 4:**
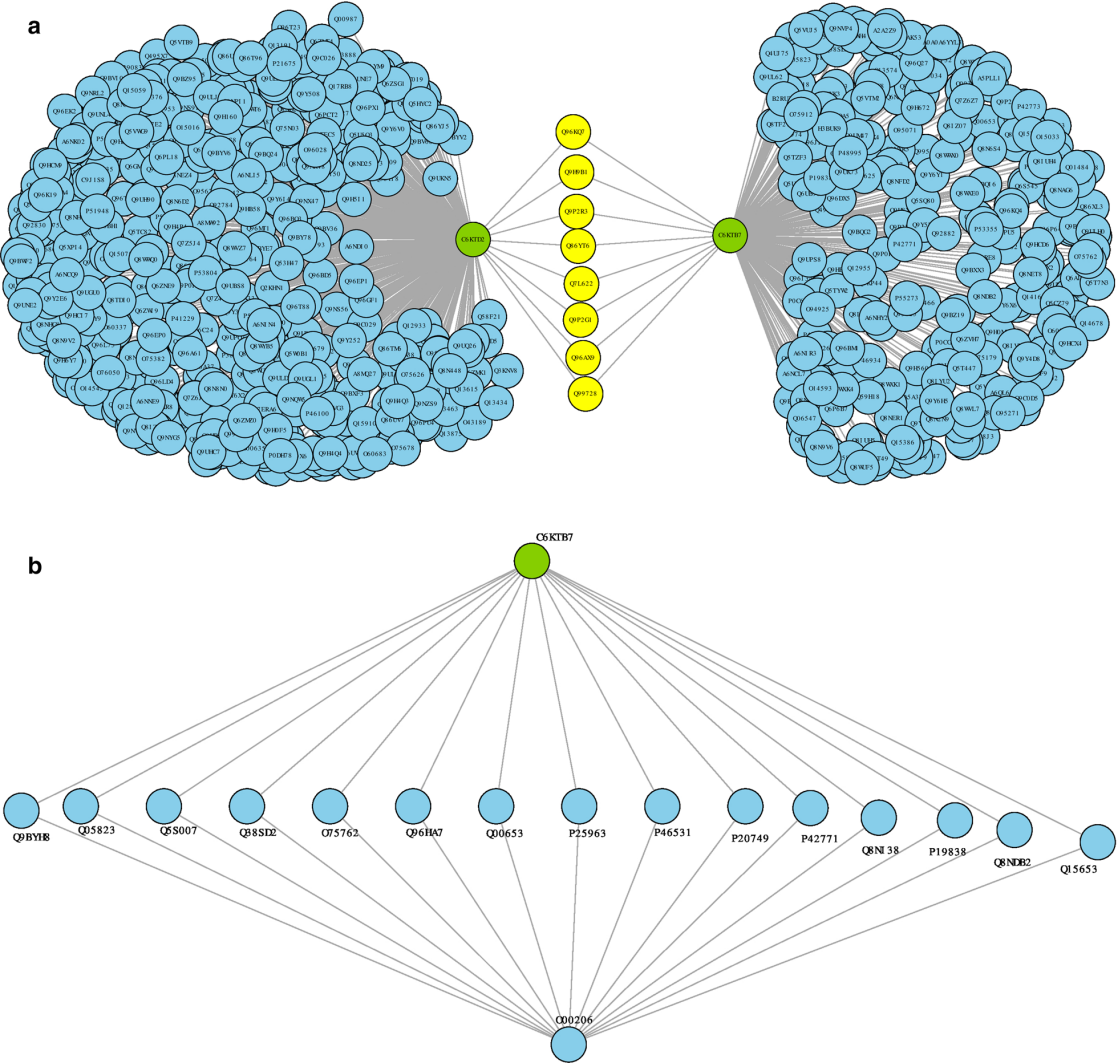

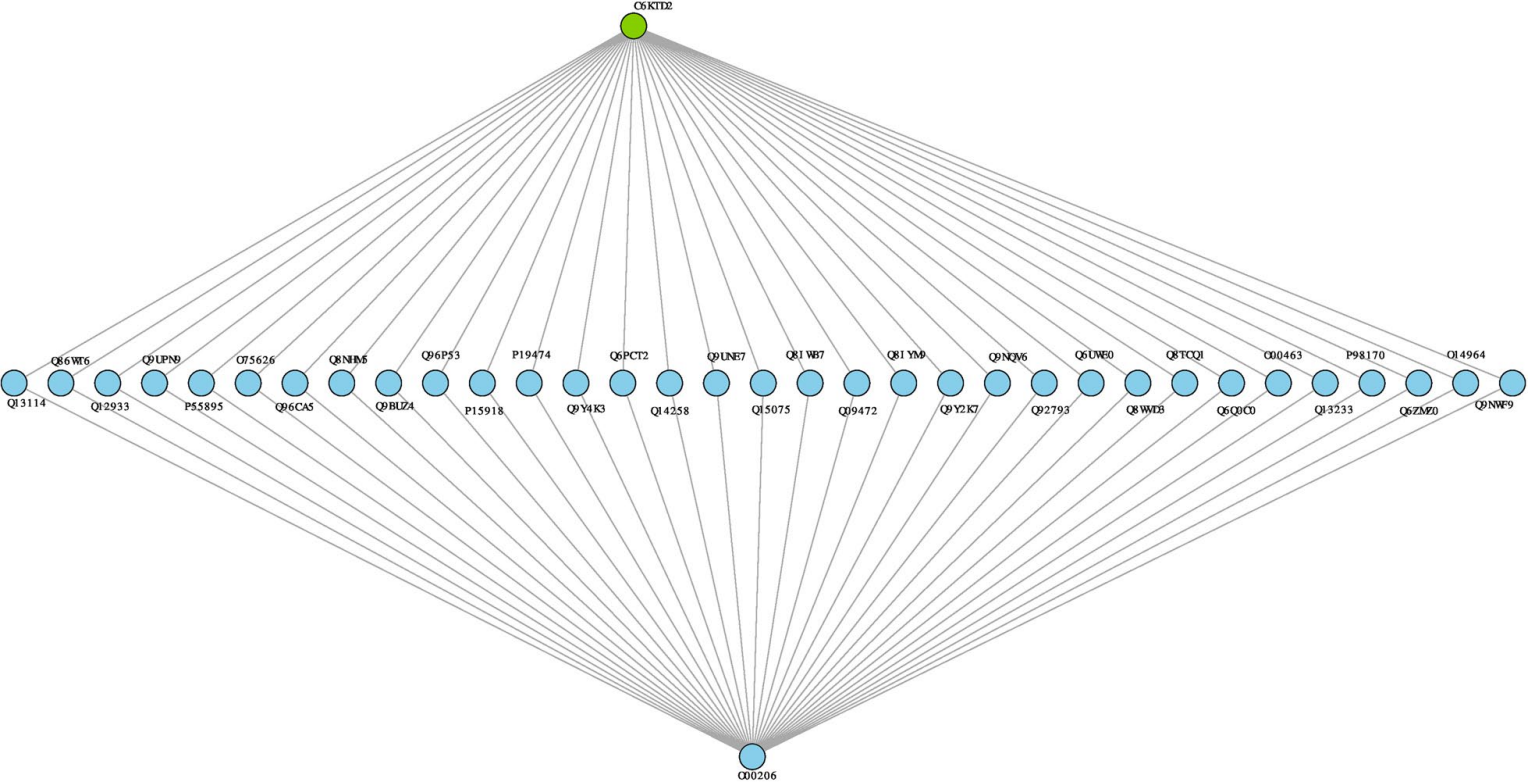
**A** Functional interactions between ***C6KTD2*** and ***C6KTB7*** subnetwork in the unified host–pathogen functional network. The shared host proteins (yellow nodes) are involved in protein ubiquitination, positive regulation of cell apoptotic process, signal transduction, regulatory processes, and histone methylation. **B** Predicted shortest path network that could influence resistance and parasite adaptiveness between ***C6KTB7*** (green node) and O00206 (bottom sky blue node) via co–targets (central sky blue nodes) in the host–pathogen network. **C** Predicted shortest path network that could influence resistance and parasite adaptiveness between ***C6KTD2*** (green node) and O00206 (bottom sky blue node) via mediators (central sky blue nodes) in the host–pathogen network

In this study, an integrative network-based framework was implemented on the various heterogeneous experimental and in silico datasets retrieved from databases and literature to assemble *Plasmodium falciparum*, human, and human-*Plasmodium falciparum* functional protein–protein interaction network. Using host-malaria GWAS summary statistics datasets, host-disease-associated genes were identified by mapping nominally significant SNPs to their associated genes. The identified genes, malaria parasite selective variants, and parasite variants under strong signature of selection were mapped onto the host and pathogen functional network respectively to identify key subnetworks. The subnetworks of each assembled network were evaluated to investigate nodes (candidate key proteins) that contribute significantly to the stability and integrity of the network. Gene annotation and enrichment analysis of the identified hub genes were performed to elucidate underlying statistically significant biological processes and pathways. Also, shortest paths analysis was performed to elucidate pathways that could account for parasite adaptiveness to host response and potential drug resistance development. From the parasite assembled functional network, the analysis performed predicted ***C6KTD2**** (SET1)* and ***C6KTB7**** (PFF1365c)* as key targets. These targets are essential at specific developmental stages of the parasite and have been reported as candidates for drug and vaccine development. The results confirm the importance of these targets. Also, the analysis (Figs. 2B and 4A) showed that these targets could be critical for combinatorial drug design. There is an accumulation of evidence that ***C6KTB7*** is a potential multi-stage target for a malaria vaccine and drug development [64–68]. ***C6KTB7*** is mainly involved in ubiquitin-protein transferase activity (GO:0004842, GO:0019787) through the protein ubiquitination and modification pathway (UPA00143). Studies have shown that many biological processes and substrates are targeted by the ubiquitin pathway such that instability or modification in ubiquitination and deubiquitination reactions influences the pathogenesis of many eukaryotic system-related diseases [65]. For instance, the dysregulation of ubiquitin ligase is associated with neurodegenerative disorders, such as Parkinson’s disease and infectious diseases including tuberculosis [66]. This is usually associated with interference with immune response. ***C6KTB7*** significantly influences the parasite’s development and malaria pathogenesis by regulating various cellular processes and pathways critical for the pathogen’s survival in the human host [69]. This phenomenon usually happens as a result of post-translational modifications within the biological system through processes such as transcriptional regulation and cell cycle progression [66]. For example, the protein is responsible for the positive regulation of DNA-templated transcription and epigenetic factors such as histone H3-K4 methylation, essential for transcription regulation [65]. Interestingly, studies have shown that inhibition of the activities of ***C6KTB7*** and the ubiquitin–proteasome system has the potential for many disease treatments including *P. falciparum* malaria [65, 68, 69]. Of note, the parasite candidate proteins are essential during specific developmental stages. For instance, Aminake et al. [68] explored the role of the proteasome of *P. falciparum* for malaria drug research and revealed ***C6KTB7*** as a component of the ubiquitin–proteasome which could serve as a promising multi-stage (liver, blood, and transmission stages of the pathogen) target, thus a supporting results presented by Chung et al. [70]. Additionally, Ponts et al. [65] showed that proteins involved in the ubiquitylation pathway including the ubiquitin ligases (E3) such as ***C6KTB7*** (PFF1365c) influence parasite virulence, thus targeting such a pathway may represent new therapeutic targets for apicomplexan parasites, such as *P. falciparum*. This suggests that inhibiting parasite adaptation to the ubiquitylation pathway and the proteins involved (including putative E3 ubiquitin-protein ligase protein PFF1365c (***C6KTB7***)) is important for malaria drug research [65, 68]. ***C6KTD2*** is a possible candidate for effective malaria vaccine development [67]. The protein plays an essential role in chromatin structure, protein domain-specific binding. and gene expression in the parasite [35, 71]. Also, it is mainly involved in the histone lysine methylation post-translational modification process (GO: 0051568) which usually involves the synergistic effect of histone-lysine methyltransferases and histone lysine demethylases [71, 72]. A gene knock-out study conducted by Jian et al. [73] revealed that ***C6KTD2*** is essential particularly during the blood stage of the parasite, thus targeting it in drug research is important. Interactome analysis on the host functional network revealed (P22301 *(IL10),* P05362 *(ICAM1),* P01375 *(TNF),* P30480 *(HLA-B),* P16284 *(PECAM1),* O00206 *(TLR4*)) as key targets. These host candidate key proteins are involved in immune response and resistance against malaria infection including severe and cerebral malaria, thus, critical targets for adjunctive and antibody-based host-directed therapy for malaria control [74–76]. Importantly, studies have shown the need to complement artemisinin derivatives with host-directed therapy involved in immune modulation to help effectively control and treat severe malaria and cerebral malaria [77]. This may contribute significantly to improve treatment efficacy, reduce disease-associated complexity, reduce malaria-associated mortality and morbidity as well as slow artemisinin resistance development. In both the parasite and host-parasite functional network, the functional interactions between hubs formed by ***C6KTD2*** and ***C6KTB7*** were identified (Fig. 2B). This finding suggests the functional relatedness of these proteins and their modularity within the parasite to jointly regulate post-translational modification processes. Having established that nodes within a cluster might be involved in the same biological process, it is, therefore, possible that these key proteins within the clusters contribute significantly to similar processes [78].

23 significantly enriched malaria-related biological processes described in (Table 3) were identified. These gene ontology groups comprised of those involved in cell immune and inflammatory responses, regulation and production of transcription factors, biosynthetic processes, cell–cell adhesion, cell signalling, and cell apoptotic processes. Positive regulation of NIK/NF-kappaB signalling (GO:0042346) process responsible for the regulation of NF-kappaB importation has been studied to be involved in immune and inflammatory responses, particularly in eukaryotic cells. Down or negative regulation of NF-kappaB has been reported to be associated with *P. falciparum*-modulated endothelium transcriptome contributing to cerebral malaria [79]. Positive regulation of the MHC class II biosynthetic process (GO:0045348) process has been shown to regulate immune response to malaria [80]. Pre-erythrocytic immunity to malaria (cerebral malaria) is linked to MHC antigens such that variations in class I and class II in these antigens contribute significantly to malaria susceptibility thus, reduced, or increased host immune response [80]. Also, other processes such as negative regulation of interferon-gamma production (GO:0032689), negative regulation of interleukin-6 production (GO:0032715), negative regulation of cytokine secretion involved in immune response (GO:0002740), and positive regulation of interferon-gamma production (GO:0032729) serves as immunological mediating processes that influence disease susceptibility by either conferring protection or influencing disease progress. Activation and regulation of NLRP3 inflammasomes, immune system receptors, controls the activation of caspase-1 and induce inflammation in response to infectious pathogens [81]. Due to their influence on a wide range of diseases, their dysfunction results in the initiation or progression of diseases. Endothelial cell apoptosis has been studied to contribute to malaria severity. For instance, haem-induced microvasculature endothelial cell apoptosis mediated by proinflammatory and proapoptotic pathways contributes significantly to severe malaria.

In addition, the pathways of immune tolerance and potential resistance development among the host and pathogen key targets were investigated by analysing the shortest paths between these genes within the host–*P. falciparum* functional network. The results showed that these shortest paths between the candidate genes or proteins are mediated by host genes involved in cell regulatory activities and general cell integrity.

Shortest path analysis further revealed human immune-related genes and pathways that could be overwhelmed by the pathogen, knowing that the pathology of malaria is immune-mediated and inflammatory response-driven. Such inhibition could result in reduced anti-inflammatory responses thus limiting the production and possible cytopathic effects of cytokines [82]. The analysis revealed potential pathways between host malaria-associated candidate key protein O00206 (Toll-like receptor 4, *TLR4*) and pathogen proteins ***C6KTB7*** (Putative E3 ubiquitin-protein ligase protein PFF1365c) and ***C6KTD2*** (Putative histone-lysine N-methyltransferase 1, *SET1*) that could account for unrestrained parasite growth and severe complications. Experimental findings have revealed that activation of TLRs induces the production of nitric oxide and synthesis of pro-inflammatory cytokines, such as TNF and IL‑1β [50, 83]. Of note, activation of TLR4 induces macrophage release of pro-inflammatory mediators, such as TNF and nitric oxide [50, 83]. It also induces the expression of adhesion molecules on endothelial cells [50]. This may suggest that PECAM1, ICAM1, and TNF are from the downstream signalling cascade generated by TLR*4* [83].

Severe malaria is associated with an increased level of pro-inflammatory cytokines (T helper 1 (Th1) cytokines) such as interleukin (IL)-12, IL-8, and interferon (IFN)-$\upgamma$ in the affected person which helps to modulate defence against the infection and limit disease progression [59, 82]. This is attributed to the fact that the severity of malaria is proportional to the flawlessness in the host inflammatory response.

*TLR4*, a pathogen-recognition receptor, detects pathogen-associated molecular mechanisms in the body and initiates immune response through activation of signalling cascades such as nuclear factorkB, mitogen-activated protein kinase (MAPK), and *Plasmodium* antigens [59]. TLR4 and its immune-related signalling pathways have been reported to contribute significantly to *P. falciparum* growth and malaria pathogenesis, such that dysregulation and dysfunction of the gene increase malaria severity, symptomatic malaria, severe malaria anaemia, and resistance in Africa [84]. This suggests that deleterious activation of *TLR4* by ***C6KTB7*** and ***C6KTD2*** will significantly contribute to parasite survival and disease susceptibility thereby causing severe pathological conditions.

Finally, a semantic similarity approach was implemented to identify 113 diseases like malaria (Additional file 8: Table S5) that facilitated the prediction of 26 potential repurposable drug hits, spanning across anti-malarials, monoclonal antibodies, immunomodulators, herbs, natural products, Janus kinase inhibitors, and thrombolytic agents, that can be computationally and experimentally modified for parasite or host-directed malaria treatment. Drug hits for each category were ranked based on the enriched similarity score. The results revealed certolizumab pegol and golimumab as hits for the monoclonal antibody category, pomalidomide for the immunomodulator category, ginseng for the herbs and natural product category, ruxolitinib for the Janus kinase inhibitors, anistreplase for the thrombolytic agent category, and chloroquine for the anti-malarial category. Additional file 9: Table S6 describes the known activity and the original therapeutic purpose of the potentially repurposable drugs identified.

## Conclusions

With the gradual emergence and spread of malaria drug resistance, considering other potential drug targets and drug candidates are essential to increase the longevity of existing drugs as well as develop alternative treatment options. In this research, integrative computational methods were leveraged to (1) predict potential drug targets for both human host and pathogen-directed drug discovery, (2) predict drug candidates that could be re-engineered for malaria treatment and, (3) identify biological processes and pathways that could be overwhelmed by the pathogen to increase within-host survival.

The analysis revealed that repurposable drugs involved in regulating host immune response to inflammatory-driven disorders and/or inhibiting residual malaria infection may enable appropriate malaria treatment. Of note, the potential to treat malaria using inhibitors or drugs that target the proteasome component and/or proteins involved in the parasite’s post-translational modification such as ***C6KTB7*** and ***C6KTD2*** have been established. However, exploring these targets for drug and vaccine development is yet to be fully achieved. Both ***C6KTD2*** and ***C6KTB7*** proteins have no crystallized structure yet, but the availability of other homologs could be explored using homology modelling approach to model the proteins. The generated homology models could be the starting point for novel drug discovery and structure-based studies to identify potential inhibitors. Additionally, the host protein targets predicted have solved structures that can be harnessed for structure-based drug discovery to identify potential inhibitors for malaria research.

In summary, the uniqueness of the integrative network framework lies in the input datasets, scoring metrics/schemes, clustering algorithm, and the criteria defined for the various analysis which translates into the findings from this study. The integrative network-based approach incorporates interologs, sequence blast interactions, and protein–protein interaction data from the literature, as well as the STRING, IntAct, MINT, and BIOGRID databases. In addition, the network approach implements a scalable hierarchical agglomerative clustering model, based on modularity optimization, to cluster the network into communities by leveraging candidate genes. This is then followed by network topology analysis to evaluate the topological features (degree, betweenness, and closeness) of the malaria candidate genes to identify hubs genes/proteins. The semantic similarity measures implemented coupled with literature evidence helped to identify diseases similar to malaria and potential repurposable drug candidates.

Like other computational approaches which need validation through further functional study, our findings presented can inform functional study for potential experimental and clinical validation. Extended computational analysis of this work would consider incorporating non-reviewed protein data, other omics level datasets, and drug-drug interaction information.

## Supplementary Information

**Additional file 1: Figure S1**. Relationship between the degree, betweenness, and closeness centrality measures in the host-parasite assembled functional network. Figures A, B and C show the relationship observed in the parasite network whereas Figures D, E, and F represent the host network. Figures A and D show that the majority of nodes are characterized by a relatively high betweenness and degree score. This depicts the small-world property of the network whereby non-neighboring nodes within the network can interact through influential nodes. Figures B and C show that lower degree nodes are usually in close interaction thus, suggesting that such nodes are involved in similar processes or pathways, thus execute the function within a smaller compartment (low-level modularity) of the system, and the effect is transmitted by central nodes with relatively higher degree and betweenness. Figures C and F suggest that signalling (flow of information) within the biological system is highly influenced by nodes with relatively high betweenness. Such nodes are characterized by relatively high degree and closeness and are known to transmit signals generated as a result of low-level modularity between nodes.

**Additional file 2: Figure S2A**. Summary results for parasite network clustering.

**Additional file 3: Figure S2B**. Summary results for host network clustering.

**Additional file 4: Table S1**. Description of various datasets and databases used for the study.

**Additional file 5: Table S2**. Malaria-associated genes were retrieved by mapping significant SNPs to the gene level. The table entails the gene’s functional network centrality scores, including betweenness, degree, and closeness.

**Additional file 6: Table S3**. Degree, closeness, and betweenness centrality score of C6KTD2 and C6KTB7 within the parasite unified functional network.

**Additional file 7: Table S4**. Degree, closeness, and betweenness centrality score for host candidate key proteins within the human functional network.

**Additional file 8: Table S5**. Predicted malaria–similar diseases identified using semantic similarity approach. ESS represents the estimated enriched similarity scores.

**Additional file 9: Table S6**. Predicted repurposable drug hits identified using semantic similarity approach.

**Additional file 10**. Supplementary method.

## Data Availability

All the scripts and data used in this manuscript are available at https://github.com/francis-agamah/Network_Analysis_Malaria. Online Mendelian Inheritance in Man (http://www.omim.org). Supplementary data and figures are available online.

## Abbreviations

WHO: World Health Organization
SNP: Single Nucleotide Polymorphism
GWAS: Genome-wide Association Study
ESS: Enriched Similarity Score
PPIN: Protein–Protein Interaction Network
ACT: Artemisinin-based Combination Therapy
