# Supplementary figures and images for: Network-driven analysis of human–Plasmodium falciparum interactome: processes for malaria drug discovery and extracting in silico targets
Source: Malar J. 2021 Oct 26;20:421. doi: 10.1186/s12936-021-03955-0 (PMC8547565; doi:10.1186/s12936-021-03955-0)

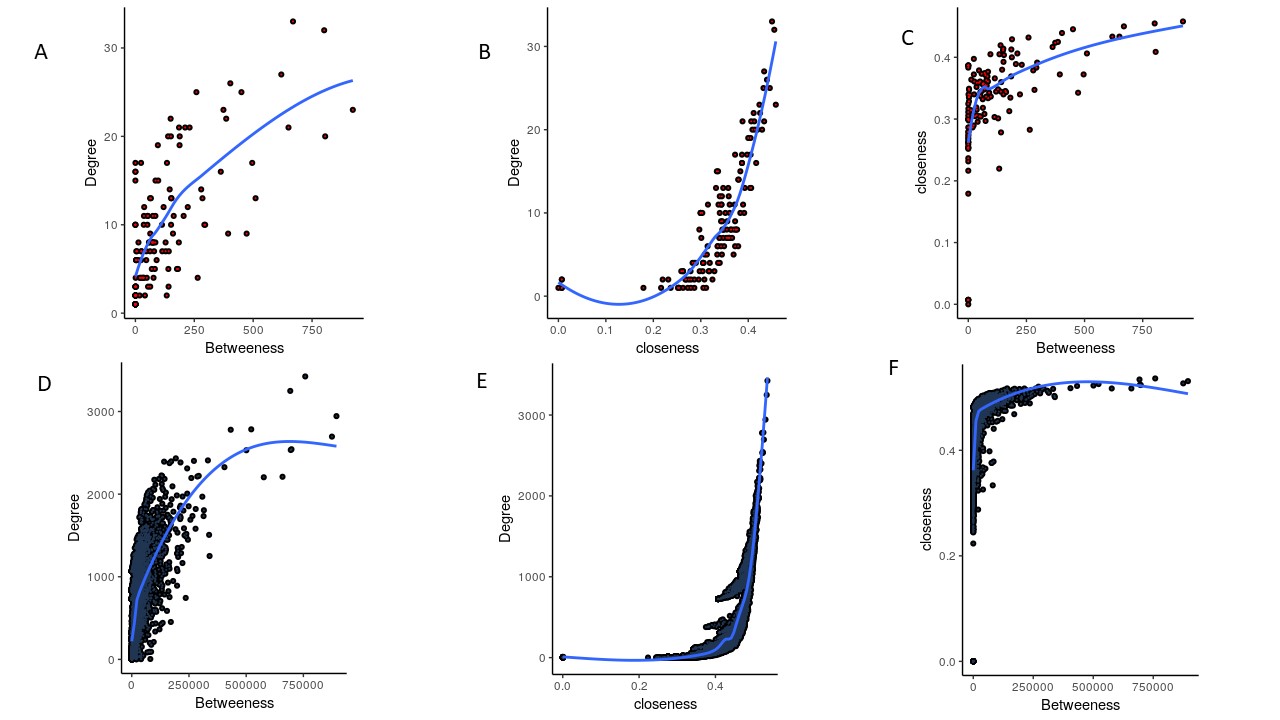

Supplement: Supplementary file 1 — Additional file 1: Figure S1. Relationship between the degree, betweenness, and closeness centrality measures in the host-parasite assembled functional network. Figures A, B and C show the relationship observed in the parasite network whereas Figures D, E, and F represent the host network. Figures A and D show that the majority of nodes are characterized by a relatively high betweenness and degree score. This depicts the small-world property of the network whereby non-neighboring nodes within the network can interact through influential nodes. Figures B and C show that lower degree nodes are usually in close interaction thus, suggesting that such nodes are involved in similar processes or pathways, thus execute the function within a smaller compartment (low-level modularity) of the system, and the effect is transmitted by central nodes with relatively higher degree and betweenness. Figures C and F suggest that signalling (flow of information) within the biological system is highly influenced by nodes with relatively high betweenness. Such nodes are characterized by relatively high degree and closeness and are known to transmit signals generated as a result of low-level modularity between nodes. [file 12936_2021_3955_MOESM1_ESM.jpg]

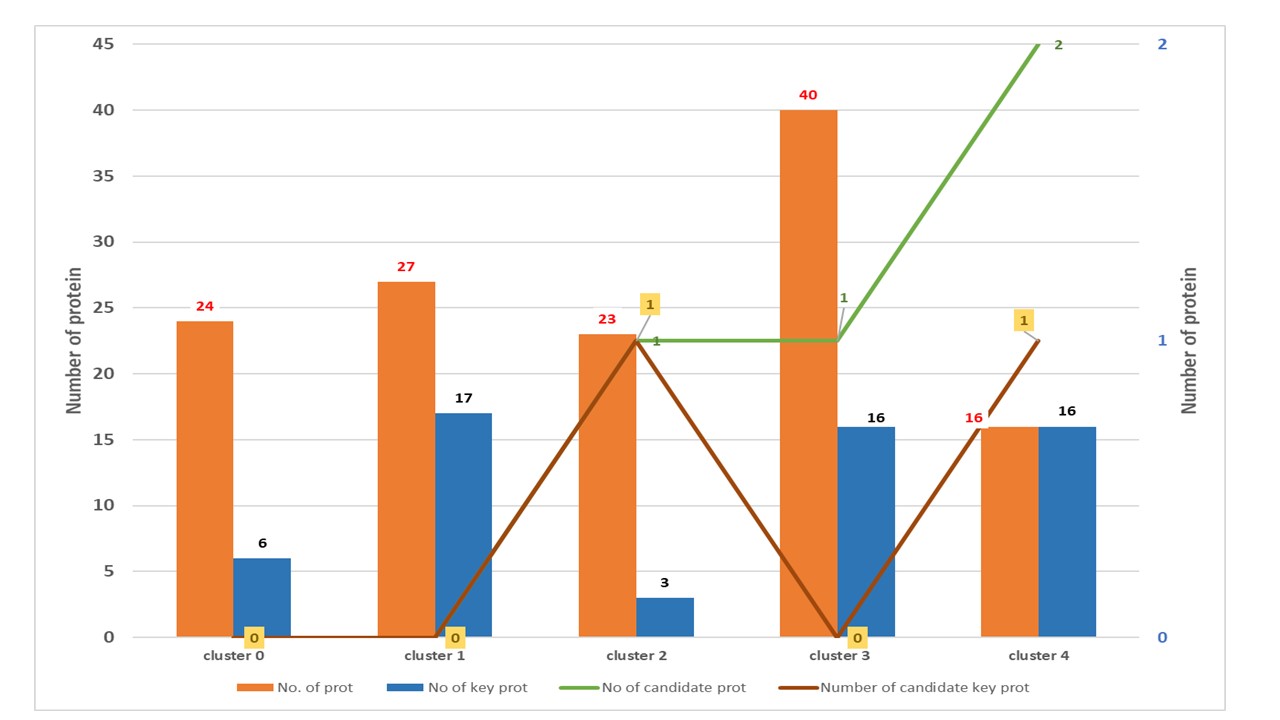

Supplement: Supplementary file 2 — Additional file 2: Figure S2A. Summary results for parasite network clustering. [file 12936_2021_3955_MOESM2_ESM.jpg]

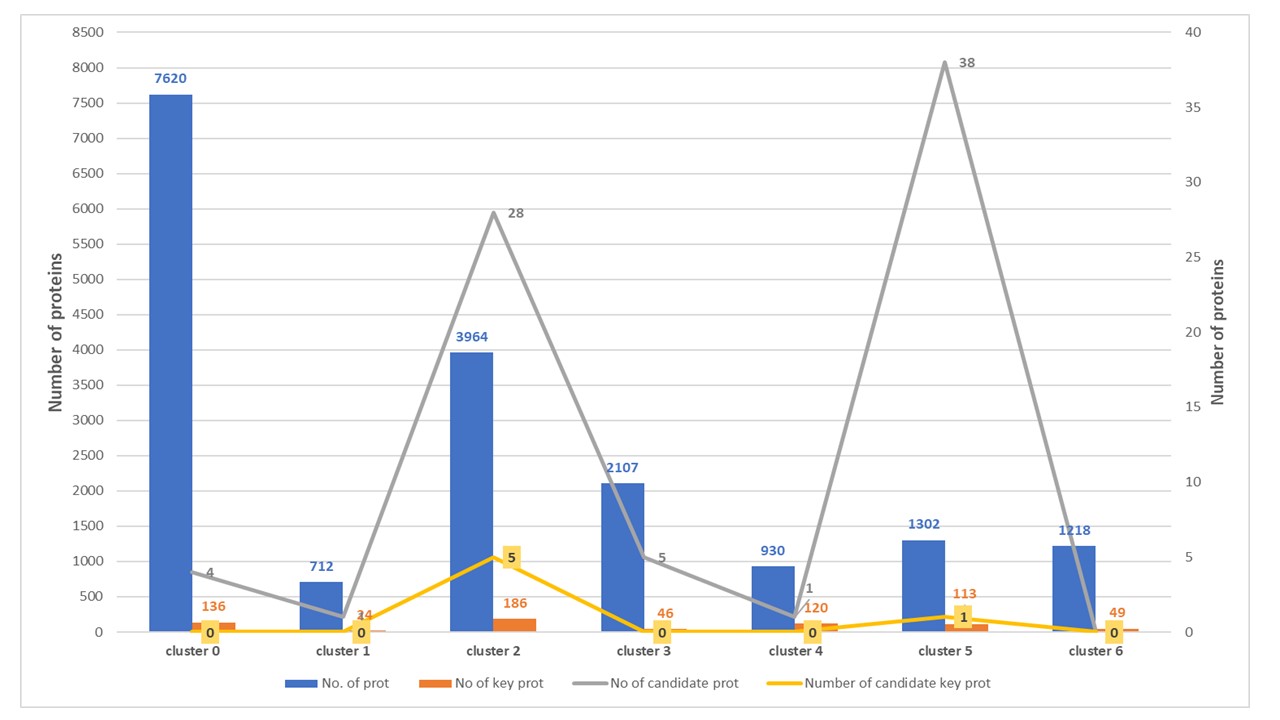

Supplement: Supplementary file 3 — Additional file 3: Figure S2B. Summary results for host network clustering. [file 12936_2021_3955_MOESM3_ESM.jpg]
