# Supplementary material for: Network-driven analysis of human–Plasmodium falciparum interactome: processes for malaria drug discovery and extracting in silico targets
Source: Malar J. 2021 Oct 26;20:421. doi: 10.1186/s12936-021-03955-0 (PMC8547565; doi:10.1186/s12936-021-03955-0)
Supplement: Supplementary file 4 — Additional file 4: Table S1. Description of various datasets and databases used for the study. [file 12936_2021_3955_MOESM4_ESM.docx]

**Table 1.** Description of various datasets and databases used for the study

| Data type | Source | Description | Reference |
| --- | --- | --- | --- |
| Human and P. falciparum Protein sequence | UniProt | Centralized resource for protein sequences  and functional information | (1) |
| Protein family and domain/signatures | Interpro | An integrative protein signature database | (2) |
| Human and P. falciparum  PPI datasets | STRING | Retrieval of functional associations  inferred from sequence and high throughput  data | (3) |
| Wuchty et al in silico Plasmodium PPI (1) | Literature | Computationally derived Plasmodium  falciparum PPIs derived using protein domains, interologs, and experimental PPIs | (4) |
| Wuchty et al experimental PPI (2) | Literature | Experimentally derived Plasmodium  falciparum PPIs derived using protein  domains | (5) |
| Wuchty experimental P. falciparum PPI (3) | Literature | Experimentally derived Plasmodium  falciparum PPIs | (6) |
| Wuchty et al experimental P. falciparum PPI (4) | Literature | Experimentally derived Plasmodium  falciparum PPIs | (7) |
| LaCount Protein  Interaction network of P. falciparum | Literature | Experimentally derived PPIs | (8) |
| Bossi and Lenner human experimental  PPIs | Literature | Experimentally derived human PPIs | (9) |
| GWAS Summary Statistics | Literature/ EGA |  | (10) |
| Functional genomics  database and tools | Reactome | Database of manually curated, peer-reviewed  pathway database of human  pathways and processes | (11) |
|  | IntAct | Protein interaction database system  and analysis tools for molecular interaction  data | (12) |
|  | MINT | Molecular interaction database of  experimentally verified PPIs mined  from scientific literatures | (13) |
|  | BioGRID | Curated biological database of PPIs,  genetic interactions, chemical interactions  and post-translational modifications | (14) |
|  | Gene Ontology  database | A classification system for annotation  of genes and gene products with  molecular function, biological process  and cellular component | (15) |
|  | KEGG Database | An integrated database of genes and  metabolic pathway information | (16) |

1. The UniProt C. UniProt: the universal protein knowledgebase. Nucleic Acids Res. 2017;45(D1):D158-D69.

2. Hunter S, Apweiler R, Attwood TK, Bairoch A, Bateman A, Binns D, et al. InterPro: the integrative protein signature database. Nucleic Acids Res. 2009;37(Database issue):D211-5.

3. von Mering C, Huynen M, Jaeggi D, Schmidt S, Bork P, Snel B. STRING: a database of predicted functional associations between proteins. Nucleic Acids Res. 2003;31(1):258-61.

4. Wuchty S, Ipsaro JJ. A draft of protein interactions in the malaria parasite P. falciparum. J Proteome Res. 2007;6(4):1461-70.

5. Wuchty S. Topology and weights in a protein domain interaction network--a novel way to predict protein interactions. BMC Genomics. 2006;7:122.

6. Wuchty S. Rich-club phenomenon in the interactome of P. falciparum--artifact or signature of a parasitic life style? PLoS One. 2007;2(3):e335.

7. Wuchty S, Adams JH, Ferdig MT. A comprehensive Plasmodium falciparum protein interaction map reveals a distinct architecture of a core interactome. Proteomics. 2009;9(7):1841-9.

8. LaCount DJ, Vignali M, Chettier R, Phansalkar A, Bell R, Hesselberth JR, et al. A protein interaction network of the malaria parasite Plasmodium falciparum. Nature. 2005;438(7064):103-7.

9. Bossi A, Lehner B. Tissue specificity and the human protein interaction network. Mol Syst Biol. 2009;5:260.

10. Malaria Genomic Epidemiology N, Band G, Rockett KA, Spencer CC, Kwiatkowski DP. A novel locus of resistance to severe malaria in a region of ancient balancing selection. Nature. 2015;526(7572):253-7.

11. Croft D, O'Kelly G, Wu G, Haw R, Gillespie M, Matthews L, et al. Reactome: a database of reactions, pathways and biological processes. Nucleic Acids Res. 2011;39(Database issue):D691-7.

12. Kerrien S, Aranda B, Breuza L, Bridge A, Broackes-Carter F, Chen C, et al. The IntAct molecular interaction database in 2012. Nucleic Acids Res. 2012;40(Database issue):D841-6.

13. Licata L, Briganti L, Peluso D, Perfetto L, Iannuccelli M, Galeota E, et al. MINT, the molecular interaction database: 2012 update. Nucleic Acids Res. 2012;40(Database issue):D857-61.

14. Chatr-Aryamontri A, Oughtred R, Boucher L, Rust J, Chang C, Kolas NK, et al. The BioGRID interaction database: 2017 update. Nucleic Acids Res. 2017;45(D1):D369-D79.

15. Harris MA, Clark J, Ireland A, Lomax J, Ashburner M, Foulger R, et al. The Gene Ontology (GO) database and informatics resource. Nucleic Acids Res. 2004;32(Database issue):D258-61.

16. Tanabe M, Kanehisa M. Using the KEGG database resource. Curr Protoc Bioinformatics. 2012;Chapter 1:Unit1 12.
