# Supplementary material for: Network-driven analysis of human–Plasmodium falciparum interactome: processes for malaria drug discovery and extracting in silico targets
Source: Malar J. 2021 Oct 26;20:421. doi: 10.1186/s12936-021-03955-0 (PMC8547565; doi:10.1186/s12936-021-03955-0)
Supplement: Supplementary file 5 — Additional file 5: Table S2. Malaria-associated genes were retrieved by mapping significant SNPs to the gene level. The table entails the gene’s functional network centrality scores, including betweenness, degree, and closeness. [file 12936_2021_3955_MOESM5_ESM.docx]

**Table 2**. Malaria-associated genes retrieved by mapping significant SNPs to gene level. The table entails the gene’s functional network centrality scores, including betweenness, degree and closeness.

| Uniprot ID | Gene name | Description | Betweenness | Degree | Closeness |
| --- | --- | --- | --- | --- | --- |
| *P23634* | *ATP2B4 [MIM: 108732]* | Plasma membrane calcium transporting ATPase 4 (PMCA4) (EC 7.2.2.10) (Matrix-remodeling associated protein 1) (Plasma membrane calcium ATPase isoform 4) (Plasma membrane calcium pump isoform 4) | 13,348.53 | 295 | 0.46096 |
| *P02818* | *BGLAP [MIM: 112260]* | Osteocalcin (Bone Gla protein) (BGP) (Gamma carboxyglutamic acid containing protein) | 17,285.08 | 344 | 0.46416 |
| *P16671* | *CD36 [MIM:* *173510]* | Platelet glycoprotein 4 (Fatty acid translocase) (FAT) (Glycoprotein IIIb) (GPIIIB) (Leukocyte differentiation antigen CD36) (PAS IV) (PAS-4) (Platelet collagen receptor) (Platelet glycoprotein IV) (GPIV) (Thrombospondin receptor) (CD antigen CD36) | 12,277.06 | 324 | 0.46485 |
| *P17927* | *CR1 [MIM: 120620]* | Complement receptor type 1 (C3b/C4b receptor) (CD antigen CD35) | 750 | 61 | 0.36270 |
| *Q16570* | *DARC [MIM: 613665]* | Atypical chemokine receptor 1 (Duffy antigen/chemokine receptor) (Fy glycoprotein)  (GpFy) (Glycoprotein D) (Plasmodium vivax receptor) (CD antigen CD234) | 2,604.91 | 143 | 0.42642 |
| *P20711* | *DDC [MIM: 107930]* | Aromatic-L-amino-acid decarboxylase (AADC) (EC 4.1.1.28) (DOPA decarboxylase) (DDC) | 8,905.58 | 219 | 0.44328 |
| *P12318* | *FCGR2A [MIM: 146790]* | Low affinity immunoglobulin gamma Fc region receptor II-a (IgG Fc receptor IIa) (CDw32) (Fc-gamma RIIa) (Fc-gamma-RIIa) (FcRII-a) (CD antigen CD32) | 24,611.42 | 1,347 | 0.48942 |
| *P08637* | *FCGR3A [MIM: 146740]* | Low affinity immunoglobulin gamma Fc region receptor III-A (CD16a antigen) (Fc-gamma RIII-alpha) (Fcgamma RIII) (Fc-gamma RIIIa) (FcRIII) (FcRIIIa) (FcR-10) (IgG Fc receptor III-2)  (CD antigen CD16a) | 833.02 | 1,085 | 0.46941 |
| *O75015* | *FCGR3B [MIM:* *610665]* | Low affinity immunoglobulin gamma Fc region receptor IIIB (Fc-gamma RIII-beta) (Fcgamma  RIII) (Fc-gamma RIIIb) (FcRIII) (FcRIIIb) (FcR-10) (IgG Fc receptor III-1) (CD antigen CD16b) | 382.27 | 1,066 | 0.46501 |
| *P0C091* | *FREM3 [MIM: 608946]* | FRAS1-related extracellular matrix protein 3 | 2,648.44 | 83 | 0.41347 |
| *P11413* | *G6PD [MIM: 305900]* | Glucose-6-phosphate 1-dehydrogenase (G6PD) (EC1.1.1.49) | 29,492.51 | 586 | 0.47380 |
| *P02724* | *GYPA [MIM: 617922]* | Glycophorin-A (MN sialoglycoprotein) (PAS-2) (Sialo glycoprotein alpha) (CD antigen CD235a) | 198.02 | 24 | 0.36143 |
| *P06028* | *GYPB [MIM:* *617923]* | Glycophorin-B (PAS-3) (SS-active sialoglycoprotein) (Sialoglycoprotein delta) (CD antigen CD235b) | 832.76 | 58 | 0.39935 |
| *P68871* | *HBB [MIM: 141900]* | Hemoglobin subunit beta (Beta-globin) (Hemoglobin beta chain) | 14,739.23 | 250 | 0.45899 |
| *P02100* | *HBE1 [MIM: 142100]* | Hemoglobin subunit epsilon (Epsilon-globin) (Hemoglobin epsilon chain) | 10,092.52 | 178 | 0.44205 |
| *P01889* | *HLA-B [MIM:* *142830]* | HLA class I histocompatibility antigen, B-7 alpha chain (MHC class I antigen B*7) | 4,058.91 | 1,079 | 0.46633 |
| *P03989* | *HLA-B* | HLA class I histocompatibility antigen, B-27 alpha chain (MHC class I antigen B*27) | 851.17 | 1,073 | 0.46468 |
| *P10319* | *HLA-B* | HLA class I histocompatibility antigen, B-58 alpha chain (Bw-58) (MHC class I antigen B*58) | 451.76 | 1,072 | 0.46419 |
| *P18463* | *HLA-B* | HLA class I histocompatibility antigen, B-37 alpha chain (MHC class I antigen B*37) | 332.13 | 1,071 | 0.46416 |
| *P18464* | *HLA-B* | HLA class I histocompatibility antigen, B-51 alpha chain (MHC class I antigen B*51) | 332.13 | 1,071 | 0.46416 |
| *P18465* | *HLA-B* | HLA class I histocompatibility antigen, B-57 alpha chain (Bw-57) (MHC class I antigen B*57) | 43,292.30 | 1,075 | 0.46424 |
| *P30460* | *HLA-B* | HLA class I histocompatibility antigen, B-8 alpha chain (MHC class I antigen B*8) | 27,355.05 | 1,073 | 0.46418 |
| *P30461* | *HLA-B* | HLA class I histocompatibility antigen, B-13 alpha chain (MHC class I antigen B*13) | 332.13 | 1,071 | 0.46416 |
| *P30462* | *HLA-B* | HLA class I histocompatibility antigen, B-14 alpha chain (MHC class I antigen B*14) | 332.13 | 1,071 | 0.46416 |
| *P30464* | *HLA-B* | HLA class I histocompatibility antigen, B-15 alpha chain (MHC class I antigen B*15) | 451.76 | 1,072 | 0.46419 |
| *P30466* | *HLA-B* | HLA class I histocompatibility antigen, B-18 alpha chain (MHC class I antigen B*18) | 332.13 | 1,071 | 0.46416 |
| *P30475* | *HLA-B* | HLA class I histocompatibility antigen, B-39 alpha chain (MHC class I antigen B*39) | 332.13 | 1,071 | 0.46416 |
| *P30479* | *HLA-B* | HLA class I histocompatibility antigen, B-41 alpha chain (Bw-41) (MHC class I antigen B*41) | 332.13 | 1,071 | 0.46416 |
| *P30480* | *HLA-B* | HLA class I histocompatibility antigen, B-42 alpha chain (MHC class I antigen B*42) | 76,201.46 | 1,330 | 0.49009 |
| *P30481* | *HLA-B* | HLA class I histocompatibility antigen, B-44 alpha chain (Bw-44) (MHC class I antigen B*44) | 352.06 | 1,072 | 0.46425 |
| *P30483* | *HLA-B* | HLA class I histocompatibility antigen, B-45 alpha chain (Bw-45) (MHC class I antigen B*45) | 352.06 | 1,072 | 0.46425 |
| *P30484* | *HLA-B* | HLA class I histocompatibility antigen, B-46 alpha chain (Bw-46) (MHC class I antigen B*46) | 451.76 | 1,072 | 0.46419 |
| *P30485* | *HLA-B* | HLA class I histocompatibility antigen, B-47 alpha chain (Bw-47) (MHC class I antigen B*47) | 332.13 | 1,071 | 0.46416 |
| *P30486* | *HLA-B* | HLA class I histocompatibility antigen, B-48 alpha chain (Bw-48) (MHC class I antigen B*48) | 332.13 | 1,071 | 0.46416 |
| *P30487* | *HLA-B* | HLA class I histocompatibility antigen, B-49 alpha chain (HLA class I histocompatibility antigen, B-21 alpha chain) (MHC class I antigen B*49) | 332.13 | 1,071 | 0.46416 |
| *P30488* | *HLA-B* | HLA class I histocompatibility antigen, B-50 alpha chain (Bw-50) (HLA class I histocompatibility  antigen, B-21 alpha chain) (MHC class I antigen B*50) | 332.13 | 1,071 | 0.46416 |
| *P30490* | *HLA-B* | HLA class I histocompatibility antigen, B-52 alpha chain (Bw-52) (HLA class I histocompatibility  antigen, B-5 alpha chain) (MHC class I antigen B*52) | 332.13 | 1,071 | 0.46416 |
| *P30491* | *HLA-B* | HLA class I histocompatibility antigen, B-53 alpha chain (Bw-53) (MHC class I antigen B*53) | 451.76 | 1,072 | 0.46419 |
| *P30492* | *HLA-B* | HLA class I histocompatibility antigen, B-54 alpha chain (Bw-54) (MHC class I antigen B*54) | 451.76 | 1,072 | 0.46419 |
| *P30493* | *HLA-B* | HLA class I histocompatibility antigen, B-55 alpha chain (Bw-55) (HLA class I histocompatibility  antigen, B-12 alpha chain) (MHC class I antigen B*55) | 451.76 | 1,072 | 0.46419 |
| *P30495* | *HLA-B* | HLA class I histocompatibility antigen, B-56 alpha chain (Bw-22) (Bw-56) (MHC class I antigen B*56) | 451.76 | 1,072 | 0.46419 |
| *P30498* | *HLA-B* | HLA class I histocompatibility antigen, B-78 alpha chain (MHC class I antigen B*78) | 332.13 | 1,071 | 0.46416 |
| *P30685* | *HLA-B* | HLA class I histocompatibility antigen, B-35 alpha chain (MHC class I antigen B*35) | 451.76 | 1,072 | 0.46419 |
| *Q04826* | *HLA-B* | HLA class I histocompatibility antigen, B-40 alpha chain (Bw-60) (MHC class I antigen B*40) | 332.13 | 1,071 | 0.46416 |
| *Q29718* | *HLA-B* | HLA class I histocompatibility antigen, B-82 alpha chain (MHC class I antigen B*82) | 332.13 | 1,071 | 0.46416 |
| *Q29836* | *HLA-B* | HLA class I histocompatibility antigen, B-67 alpha chain (MHC class I antigen B*67) | 332.13 | 1,071 | 0.46416 |
| *Q29940* | *HLA-B* | HLA class I histocompatibility antigen, B-59 alpha chain (MHC class I antigen B*59) | 451.76 | 1,072 | 0.46419 |
| *Q31610* | *HLA-B* | HLA class I histocompatibility antigen, B-81 alpha chain (B’DT) (MHC class I antigen B*81) | 332.13 | 1,071 | 0.46416 |
| *Q31612* | *HLA-B* | HLA class I histocompatibility antigen, B-73 alpha chain (MHC class I antigen B*73) | 32,036.80 | 1,283 | 0.49117 |
| *Q95365* | *HLA-B* | HLA class I histocompatibility antigen, B-38 alpha chain (Bw-4) (MHC class I antigen B*38) | 345.40 | 1,073 | 0.46418 |
| *Q30154* | *HLADRB5 [MIM: 604776]* | HLA class II histocompatibility antigen, DR beta 5 chain (DR beta-5) (DR2-beta- 2) (Dw2) (MHC class II antigen DRB5) | 26,778.34 | 1,290 | 0.48645 |
| *P09601* | *HMOX1 [MIM: 141250]* | Heme oxygenase 1 (HO-1) (EC 1.14.14.18) | 27,916.14 | 515 | 0.47913 |
| *P00738* | *HP [140100]* | Haptoglobin (Zonulin) [Cleaved into: Haptoglobin alpha chain; Haptoglobin beta chain] | 44,329.89 | 554 | 0.47580 |
| *P05362* | *ICAM1 [MIM: 147840]* | Intercellular adhesion molecule 1 (ICAM-1) (Major group rhinovirus receptor) (CD antigen CD54) | 68,177.95 | 1,690 | 0.50307 |
| *P01562* | *IFNA1* | Interferon alpha-1/13 (IFN alpha- 1/13) (Interferon alpha- D) (LeIF D) | 3,160.95 | 255 | 0.44405 |
| *P22301* | *IL10 [MIM: 124092]* | Interleukin-10 (IL-10) (Cytokine synthesis inhibitory factor) (CSIF) | 60,689.17 | 1,027 | 0.49031 |
| *P35225* | *IL13 [MIM: 147683]* | Interleukin-13 (IL-13) | 16,060.33 | 589 | 0.46863 |
| *P01584* | *IL1B [MIM: 147720]* | Interleukin-1 beta (IL-1 beta) (Catabolin) | 43,577.22 | 866 | 0.48631 |
| *P18510* | *IL1RN [MIM: 147679]* | Interleukin-1 receptor antagonist protein (IL-1RN) (IL-1ra) (IRAP) (ICIL-1RA) (IL1 inhibitor)  (Anakinra) | 4,992.78 | 340 | 0.45408 |
| *P05112* | *IL4 [MIM: 147780]* | Interleukin-4 (IL-4) (B-cell stimulatory factor 1) (BSF-1) (Binetrakin) (Lymphocyte stimulatory factor 1) (Pitrakinra) | 50,896.72 | 899 | 0.48677 |
| *O15327* | *INPP4B [MIM: 607494]* | Type II inositol 3,4-bisphosphate 4-phosphatase (EC 3.1.3.66) (Inositol polyphosphate 4-phosphatase type II) | 6,224.22 | 263 | 0.45068 |
| *P10914* | *IRF1 [MIM: 147575]* | Interferon regulatory factor 1 (IRF-1) | 24,455.19 | 674 | 0.48029 |
| *Q96A59* | *MARVELD [MIM: 614094]* | MARVEL domain-containing protein 3 | 2,526.89 | 84 | 0.39215 |
| *P11226* | *MBL2 [MIM: 154545]* | Mannose-binding protein C (MBP-C) (Collectin-1) (MBP1) (Mannan-binding protein) Mannose-binding lectin) | 13,984.13 | 356 | 0.44608 |
| *P03971* | *MIF [MIM: 600957]* | Muellerian-inhibiting factor (Anti-Muellerian hormone) (AMH) (Muellerian-inhibiting  substance) (MIS) | 9,561.06 | 279 | 0.45369 |
| *P14174* | *MIF [MIM: 153620]* | Macrophage migration inhibitory factor (MIF) (EC 5.3.2.1) (Glycosylation inhibiting factor) (GIF) (L-dopachrome isomerase) (L-dopachrome tautomerase) (EC 5.3.3.12) (Phenylpyruvate  tautomerase) | 4,797.51 | 241 | 0.45795 |
| *P11055* | *MYH3 [MIM: 160720]* | Myosin-3 (Muscle embryonic myosin heavy chain) (Myosin heavy chain 3) (Myosin heavy  chain, fast skeletal muscle, embryonic) (SMHCE) | 26,135.36 | 1,122 | 0.49250 |
| *P60321* | *NANOS2 [MIM: 608228]* | Nanos homolog 2 (NOS-2) | 3,112.42 | 123 | 0.42336 |
| *P35228* | *NOS2,*  *NOS2A [MIM: 163730]* | Nitric oxide synthase, inducible (EC 1.14.13.39) (Hepatocyte NOS) (HEP-NOS) (Inducible NO synthase) (Inducible NOS) (iNOS) (NOS type II) (Peptidyl-cysteine Snitrosylase NOS2) | 14,718.65 | 425 | 0.47056 |
| *Q9H2C8* | *OR51V1* | Olfactory receptor 51V1 (Odorant receptor HOR3’beta1) (Olfactory receptor 51A12) (Olfactory receptor OR11-36) | 2,857.04 | 755 | 0.42798 |
| *P16284* | *PECAM1 [MIM:* *173445]* | Platelet endothelial cell adhesion molecule (PECAM-1) (EndoCAM) (GPIIA’) (PECA1) (CD antigen CD31) | 63,425.28 | 1,634 | 0.49978 |
| *P28065* | *PSMB9 [MIM: 177045]* | Proteasome subunit beta type-9 (EC 3.4.25.1) (Low molecular mass protein 2) (Macropain chain 7) (Multicatalytic endopeptidase complex chain 7) (Proteasome chain 7) (Proteasome subunit beta-1i) (Really interesting new gene 12 protein) | 13,964.38 | 417 | 0.46688 |
| *O75880* | *SCO1 [MIM:* *603644]* | Protein SCO1 homolog, mitochondrial | 20,845.03 | 391 | 0.44647 |
| *O00206* | *TLR4 [MIM: 603030]* | Toll-like receptor 4 (hToll) (CD antigen CD284) | 111,490.44 | 1,360 | 0.50077 |
| *Q9NR96* | *TLR9 [MIM:* *605474]* | Toll-like receptor 9 (CD antigen CD289) | 25,906.76 | 869 | 0.48774 |
| *P01375* | *TNF [MIM: 191160]* | Tumor necrosis factor (Cachectin) (TNF-alpha) (Tumor necrosis factor ligand superfamily member 2) (TNFa) [Cleaved into: Tumor necrosis factor, membrane form (N-terminal fragment) (NTF); Intracellular domain 1 (ICD1); Intracellular domain 2 (ICD2); C-domain 1; | 315,200.15 | 1,805 | 0.50908 |
| *Q13829* | *TNFAIP1 [MIM:* *191161]* | BTB/POZ domain-containing adapter for CUL3-mediated RhoA degradation protein 2  (hBACURD2) (BTB/POZ domain-containing protein TNFAIP1) (Protein B12) (Tumor necrosis tor, alpha-induced protein 1, endothelial) | 7,341.54 | 278 | 0.44825 |
| *P0DSE2* | *TRB* | M1-specific T cell receptor beta chain (TR beta chain TRBV19*01J2S7*01C*02) |  |  |  |
| *Q8NB14* | *USP38 [MIM: 618322]* | Ubiquitin carboxyl-terminal hydrolase 38 (EC 3.4.19.12) (Deubiquitinating enzyme 38) (HP43.8KD) (Ubiquitin thioesterase 38) (Ubiquitin specific- processing protease 38) | 5,893.34 | 244 | 0.43786 |
