# Supplementary material for: Network-driven analysis of human–Plasmodium falciparum interactome: processes for malaria drug discovery and extracting in silico targets
Source: Malar J. 2021 Oct 26;20:421. doi: 10.1186/s12936-021-03955-0 (PMC8547565; doi:10.1186/s12936-021-03955-0)
Supplement: Supplementary file 6 — Additional file 6: Table S3. Degree, closeness, and betweenness centrality score of C6KTD2 and C6KTB7 within the parasite unified functional network. [file 12936_2021_3955_MOESM6_ESM.docx]

Supplementary Table 3: Degree, closeness, and betweenness centrality score of *C6KTD2* and *C6KTB7* within the parasite unified functional network

| **Uniprot ID** | **Gene name** | **Degree** | **Closeness** | **Betweenness** |
| --- | --- | --- | --- | --- |
| *C6KTD2* | *SET1* | 13 | 0.40623 | 510.18 |
| *C6KTB7* | *PFF1365c* | 13 | 0.34726 | 284.70 |
