# Supplementary material for: Network-driven analysis of human–Plasmodium falciparum interactome: processes for malaria drug discovery and extracting in silico targets
Source: Malar J. 2021 Oct 26;20:421. doi: 10.1186/s12936-021-03955-0 (PMC8547565; doi:10.1186/s12936-021-03955-0)
Supplement: Supplementary file 7 — Additional file 7: Table S4. Degree, closeness, and betweenness centrality score for host candidate key proteins within the human functional network. [file 12936_2021_3955_MOESM7_ESM.docx]

Supplementary Table 4: Degree, closeness, and betweenness centrality score for host candidate key proteins within the human functional network

| **Uniprot-ID** | **Gene name** | **Degree** | **Closeness** | **Betweenness** |
| --- | --- | --- | --- | --- |
| *P01375* | *TNF* | 1805 | 0.50908 | 315200.15 |
| *P05362* | *ICAM1* | 1690 | 0.50307 | 68177.95 |
| *P16284* | *PECAM1* | 1634 | 0.49978 | 63425.28 |
| *O00206* | *TLR4* | 1360 | 0.50077 | 111490.44 |
| *P30480* | *HLA-B* | 1330 | 0.49009 | 76201.46 |
| *P22301* | *IL10* | 1027 | 0.49031 | 60689.17 |
