# Supplementary material for: Network-driven analysis of human–Plasmodium falciparum interactome: processes for malaria drug discovery and extracting in silico targets
Source: Malar J. 2021 Oct 26;20:421. doi: 10.1186/s12936-021-03955-0 (PMC8547565; doi:10.1186/s12936-021-03955-0)
Supplement: Supplementary file 8 — Additional file 8: Table S5. Predicted malaria–similar diseases identified using semantic similarity approach. ESS represents the estimated enriched similarity scores. [file 12936_2021_3955_MOESM8_ESM.docx]

**Table 5**. Predicted malaria–similar diseases identified using semantic similarity approach. ESS represents the estimated enriched similarity scores.

| Disease-ID | Disease Name | No. disease-target associated pathways | No. of common pathways | Enriched Similarity Score (ESS) | Kappa measure | Jaccard measure |
| --- | --- | --- | --- | --- | --- | --- |
| C0001175 | Acquired Immunodeficiency Syndrome [MIM: 609423] | 226 | 69 | 0.54266 | 0.0 | 0.30531 |
| C0002871 | Anemia [MIM: 300751] | 277 | 69 | 0.51979 | 0.0 | 0.24909 |
| C0002873 | Anemia of chronic disease | 140 | 66 | 0.51902 | -0.04288 | 0.47143 |
| C0002874 | Aplastic Anemia [MIM: 609135] | 223 | 68 | 0.51064 | -0.00899 | 0.30493 |
| C0002893 | Refractory anemias | 248 | 69 | 0.49309 | 0.0 | 0.27823 |
| C0002895 | Anemia, Sickle Cell [MIM: 603903] | 255 | 68 | 0.55156 | -0.00786 | 0.26667 |
| C0003123 | Anorexia [MIM: 606788] | 200 | 68 | 0.52716 | -0.01002 | 0.34 |
| C0003864 | Arthritis | 270 | 69 | 0.50061 | 0.0 | 0.25556 |
| C0006111 | Brain Diseases | 250 | 69 | 0.52636 | 0.0 | 0.276 |
| C0006118 | Brain Neoplasms | 277 | 69 | 0.51262 | 0.0 | 0.24909 |
| C0006287 | Bronchopulmonary Dysplasia | 237 | 69 | 0.5246 | 0.0 | 0.29114 |
| C0007785 | Cerebral Infarction [MIM: 601367] | 263 | 69 | 0.50857 | 0.0 | 0.26236 |
| C0007786 | Brain Ischemia | 237 | 69 | 0.52724 | 0.0 | 0.29114 |
| C0007789 | Cerebral Palsy [MIM: 605388] | 201 | 68 | 0.51322 | -0.00997 | 0.33831 |
| C0007847 | Malignant tumor of cervix | 283 | 69 | 0.51019 | 0.0 | 0.24382 |
| Continued on next page | | | | | | |
| Disease-ID | **Disease Name** | **No. disease-target associated pathways** | **No. of common pathways** | **Enriched Similarity Score (ESS)** | **Kappa measure** | **Jaccard measure** |
| C0011311 | Dengue Fever [MIM: 614371] | 196 | 69 | 0.53769 | 0.0 | 0.35204 |
| C0015672 | Fatigue | 261 | 69 | 0.52095 | 0.0 | 0.26437 |
| C0015674 | Chronic Fatigue Syndrome | 148 | 69 | 0.56246 | 0.0 | 0.46622 |
| C0015967 | Fever [MIM: 142680] | 228 | 69 | 0.51222 | 0.0 | 0.30263 |
| C0018621 | Hay fever | 178 | 69 | 0.55729 | 0.0 | 0.38764 |
| C0019101 | Hemorrhagic Fever with Renal Syndrome | 109 | 68 | 0.54928 | -0.01823 | 0.62385 |
| C0019158 | Hepatitis | 268 | 69 | 0.50801 | 0.0 | 0.25746 |
| C0019159 | Hepatitis A [MIM: 606518] | 253 | 69 | 0.52661 | 0.0 | 0.27273 |
| C0019187 | Hepatitis, Alcoholic | 178 | 68 | 0.55737 | -0.01126 | 0.38202 |
| C0019189 | Hepatitis, Chronic | 212 | 69 | 0.53011 | 0.0 | 0.32547 |
| C0019193 | Hepatitis, Toxic | 253 | 69 | 0.52166 | 0.0 | 0.27273 |
| C0019196 | Hepatitis C [MIM: 609532] | 285 | 69 | 0.51194 | 0.0 | 0.24211 |
| C0019207 | Hepatoma, Morris | 200 | 66 | 0.53196 | -0.03021 | 0.33 |
| C0019208 | Hepatoma, Novikoff | 200 | 66 | 0.53286 | -0.03021 | 0.33 |
| C0020542 | Pulmonary Hypertension [MIM: 178600] | 214 | 69 | 0.52169 | 0.0 | 0.32243 |
| C0021400 | Influenza [MIM: 614680] | 269 | 69 | 0.50113 | 0.0 | 0.25651 |
| C0023267 | Fibroid Tumor | 248 | 69 | 0.52817 | 0.0 | 0.27823 |
| Continued on next page | | | | | | |
| Disease-ID | **Disease Name** | **No. disease-target associated pathways** | **No. of common pathways** | **Enriched Similarity Score (ESS)** | **Kappa measure** | **Jaccard measure** |
| C0023290 | Leishmaniasis, Visceral [MIM: 608207] | 182 | 69 | 0.54409 | 0.0 | 0.37912 |
| C0023440 | Acute Erythroblastic Leukemia | 236 | 68 | 0.49545 | -0.00849 | 0.28814 |
| C0027497 | Nausea | 194 | 67 | 0.52302 | -0.02071 | 0.34536 |
| C0027498 | Nausea and vomiting | 219 | 67 | 0.52054 | -0.01836 | 0.30594 |
| C0027540 | Necrosis | 177 | 66 | 0.53155 | -0.03411 | 0.37288 |
| C0034063 | Pulmonary Edema [MIM: 178400] | 186 | 67 | 0.53161 | -0.02160 | 0.36022 |
| C0034067 | Pulmonary Emphysema [MIM: 210050] | 227 | 69 | 0.52845 | 0.0 | 0.30396 |
| C0034069 | Pulmonary Fibrosis [MIM: 178500] | 254 | 69 | 0.507 | 0.0 | 0.27165 |
| C0035220 | Respiratory Distress Syndrome, Newborn [MIM: 267450] | 193 | 67 | 0.53741 | -0.02082 | 0.34715 |
| C0035222 | Respiratory Distress Syndrome, Adult | 238 | 69 | 0.54066 | 0.0 | 0.28992 |
| C0035235 | Respiratory Syncytial Virus Infections | 183 | 69 | 0.54611 | 0.0 | 0.37705 |
| C0035242 | Respiratory Tract Diseases | 171 | 67 | 0.54095 | -0.02348 | 0.39181 |
| C0035436 | Rheumatic Fever [MIM: 268240] | 166 | 68 | 0.51759 | -0.01207 | 0.40964 |
| C0036205 | Sarcoidosis, Pulmonary [MIM: 181000] | 152 | 69 | 0.53268 | 0.0 | 0.45395 |
| C0036974 | Shock | 106 | 66 | 0.5684 | -0.05525 | 0.62264 |
| C1145670 | Respiratory Failure | 208 | 67 | 0.53727 | -0.01932 | 0.32212 |
| Continued on next page | | | | | | |
| Disease-ID | **Disease Name** | **No. disease-target associated pathways** | **No. of common pathways** | **Enriched Similarity Score (ESS)** | **Kappa measure** | **Jaccard measure** |
| C0036983 | Septic Shock | 113 | 68 | 0.5668 | -0.01761 | 0.60177 |
| C0038436 | Post-Traumatic Stress Disorder | 203 | 69 | 0.53531 | 0.0 | 0.33990 |
| C0040034 | Thrombocytopenia [MIM: 313900] | 269 | 69 | 0.51614 | 0.0 | 0.25651 |
| C0041228 | African Trypanosomiasis | 134 | 67 | 0.57142 | -0.02982 | 0.5 |
| C0041296 | Tuberculosis [MIM: 607948] | 277 | 69 | 0.50244 | 0.0 | 0.24909 |
| C0041327 | Tuberculosis, Pulmonary | 207 | 69 | 0.54692 | 0.0 | 0.33333 |
| C0041466 | Typhoid Fever | 125 | 67 | 0.56711 | -0.03188 | 0.536 |
| C0042721 | Viral hepatitis | 168 | 67 | 0.51131 | -0.02389 | 0.39881 |
| C0085293 | Hepatitis E | 121 | 68 | 0.55996 | -0.01648 | 0.56198 |
| C0085605 | Liver Failure [MIM: 613070] | 239 | 69 | 0.5282 | 0.0 | 0.28870 |
| C0085742 | Injuries, Acute Brain | 157 | 67 | 0.54269 | -0.02555 | 0.42675 |
| C0086404 | Experimental Hepatoma | 199 | 66 | 0.53361 | -0.03036 | 0.33166 |
| C0086565 | Liver Dysfunction | 250 | 69 | 0.49199 | 0.0 | 0.276 |
| C0151332 | Active tuberculosis | 114 | 69 | 0.56211 | 0.0 | 0.60526 |
| C0152171 | Idiopathic pulmonary hypertension [MIM: 178600] | 202 | 67 | 0.48326 | -0.01989 | 0.33168 |
| C1719672 | Severe Sepsis | 194 | 69 | 0.53222 | 0.0 | 0.35567 |
| C0272945 | Brain Lacerations | 157 | 67 | 0.54269 | -0.02555 | 0.42675 |
| Continued on next page | | | | | | |
| Disease-ID | **Disease Name** | **No. disease-target associated pathways** | **No. of common pathways** | **Enriched Similarity Score (ESS)** | **Kappa measure** | **Jaccard measure** |
| C0155728 | Other specified transient cerebral ischemias | 212 | 68 | 0.5116 | -0.00946 | 0.32075 |
| C0206624 | Hepatoblastoma [MIM: 114550] | 253 | 69 | 0.52569 | 0.0 | 0.27273 |
| C0206754 | Neuroendocrine Tumors | 235 | 68 | 0.51095 | -0.00853 | 0.28936 |
| C0220620 | Gastrointestinal Carcinoid Tumor [MIM: 114900] | 218 | 68 | 0.49072 | -0.00919 | 0.31193 |
| C0220650 | Metastatic malignant neoplasm to brain | 220 | 68 | 0.52063 | -0.00911 | 0.30909 |
| C0221505 | Lesion of brain | 191 | 69 | 0.53327 | 0.0 | 0.36126 |
| C0231528 | Myalgia | 167 | 68 | 0.53991 | -0.01199 | 0.40719 |
| C0235946 | Cerebral atrophy | 268 | 69 | 0.48964 | 0.0 | 0.25746 |
| C0241910 | Hepatitis, Autoimmune | 204 | 69 | 0.52297 | 0.0 | 0.33824 |
| C0242584 | Autoimmune thrombocytopenia | 160 | 68 | 0.54667 | -0.01252 | 0.425 |
| C0242966 | Systemic Inflammatory Response Syndrome | 167 | 69 | 0.5554 | 0.0 | 0.41317 |
| C0243026 | Sepsis | 280 | 69 | 0.5082 | 0.0 | 0.24643 |
| C0270611 | Brain Injuries | 157 | 67 | 0.54269 | -0.02555 | 0.42675 |
| C0271650 | Impaired glucose tolerance | 273 | 69 | 0.52643 | 0.0 | 0.25275 |
| C0271907 | Acquired aplastic anemia [MIM: 609135] | 153 | 66 | 0.53914 | -0.03935 | 0.43137 |
| C3241937 | Nonalcoholic Steatohepatitis [MIM: 613282] | 256 | 68 | 0.52614 | -0.00783 | 0.26563 |
| C0282687 | Hemorrhagic Fever, Ebola | 112 | 67 | 0.56975 | -0.03533 | 0.59821 |
| Continued on next page | | | | | | |
| Disease-ID | **Disease Name** | **No. disease-target associated pathways** | **No. of common pathways** | **Enriched Similarity Score (ESS)** | **Kappa measure** | **Jaccard measure** |
| C3203102 | Idiopathic pulmonary arterial hypertension [MIM: 178600] | 251 | 69 | 0.50683 | 0.0 | 0.27490 |
| C3469521 | Fanconi anemia, Complementation group A (disorder) [MIM: 227650] | 246 | 69 | 0.52568 | 0.0 | 0.28049 |
| C0375023 | Respiratory syncytial virus (RSV) infection in conditions classified elsewhere and of unspecified site | 219 | 69 | 0.5294 | 0.0 | 0.31507 |
| C0452047 | Brain Injuries, Focal | 157 | 67 | 0.54269 | -0.02555 | 0.42675 |
| C0521158 | Recurrent tumor | 258 | 67 | 0.53361 | -0.01558 | 0.25969 |
| C0524909 | Hepatitis B, Chronic [MIM: 610424] | 230 | 69 | 0.51533 | 0.0 | 0.3 |
| C0524910 | Hepatitis C, Chronic [MIM: 609532] | 258 | 69 | 0.52971 | 0.0 | 0.26744 |
| C0598935 | Tumor Initiation | 242 | 68 | 0.51708 | -0.00829 | 0.28099 |
| C0600327 | Toxic Shock Syndrome | 135 | 67 | 0.53981 | -0.02961 | 0.49629 |
| C0740391 | Middle Cerebral Artery Occlusion | 204 | 68 | 0.51866 | -0.00983 | 0.33333 |
| C0740392 | Infarction, Middle Cerebral Artery | 209 | 68 | 0.54169 | -0.00959 | 0.32536 |
| C0751690 | Malignant Peripheral Nerve Sheath Tumor | 212 | 67 | 0.51253 | -0.01896 | 0.31604 |
| C0751955 | Brain Infarction | 166 | 67 | 0.52277 | -0.02418 | 0.40361 |
| C0917798 | Cerebral Ischemia | 252 | 69 | 0.50236 | 0.0 | 0.27381 |
| C0264408 | Childhood asthma [MIM: 600807] | 208 | 69 | 0.54101 | 0.0 | 0.33173 |
| C3263723 | Traumatic injury | 104 | 67 | 0.54475 | -0.03776 | 0.64423 |
| Continued on next page | | | | | | |
| Disease-ID | **Disease Name** | **No. disease-target associated pathways** | **No. of common pathways** | **Enriched Similarity Score (ESS)** | **Kappa measure** | **Jaccard measure** |
| C0917996 | Cerebral Aneurysm [MIM: 210050] | 149 | 69 | 0.57101 | 0.0 | 0.46309 |
| C0948008 | Ischemic stroke [MIM: 601367] | 258 | 69 | 0.51378 | 0.0 | 0.26744 |
| C1175175 | Severe Acute Respiratory Syndrome | 181 | 68 | 0.55008 | -0.01107 | 0.37569 |
| C1262760 | Hepatitis, Drug-Induced | 253 | 69 | 0.52172 | 0.0 | 0.27273 |
| C1275126 | TNF receptor-associated periodic fever syndrome (TRAPS) [MIM: 142680] | 141 | 67 | 0.55724 | -0.02839 | 0.47528 |
| C1282496 | Metastasis from malignant tumor of prostate | 228 | 68 | 0.51473 | -0.00879 | 0.29825 |
| C1290398 | Cerebral arterial aneurysm | 173 | 69 | 0.55418 | 0.0 | 0.39884 |
| C1336708 | Testicular Germ Cell Tumor [MIM: 273300] | 207 | 68 | 0.51821 | -0.00969 | 0.32850 |
| C1512409 | Hepatocarcinogenesis | 272 | 68 | 0.52216 | -0.00737 | 0.25 |
| C1519666 | Tumor-Associated Vasculature | 140 | 66 | 0.54477 | -0.04288 | 0.47143 |
| C1519670 | Tumor Angiogenesis | 265 | 69 | 0.51521 | 0.0 | 0.26038 |
| C1519680 | Tumor Immunity | 192 | 68 | 0.54724 | -0.01044 | 0.35417 |
| C1658953 | tumor vasculature | 196 | 68 | 0.52328 | -0.01023 | 0.34694 |
| C1800706 | Idiopathic Pulmonary Fibrosis [MIM: 178500] | 245 | 69 | 0.54026 | 0.0 | 0.28163 |
| C1857276 | Trichohepatoenteric Syndrome [MIM: 222470] | 244 | 68 | 0.48569 | -0.00822 | 0.27869 |
|  | | | | | | |
