# Supplementary material for: Network-driven analysis of human–Plasmodium falciparum interactome: processes for malaria drug discovery and extracting in silico targets
Source: Malar J. 2021 Oct 26;20:421. doi: 10.1186/s12936-021-03955-0 (PMC8547565; doi:10.1186/s12936-021-03955-0)
Supplement: Supplementary file 9 — Additional file 9: Table S6. Predicted repurposable drug hits identified using semantic similarity approach. [file 12936_2021_3955_MOESM9_ESM.docx]

**Table 6:** Predicted repurposable drug hits identified using semantic similarity approach. Drug information collected from DrugBank ( www.drugbank.ca/)

| Drug Category | Drug Bank ID | Drug Name | Original therapeutic purpose(s) | Target | Known activity | Enriched Similarity Score (ESS) | Non-redundant Enriched Similarity Score (NRSS) | Redundant similarity score (RSS) |
| --- | --- | --- | --- | --- | --- | --- | --- | --- |
| Antimalarial | DB00608 | Chloroquine | Rheumatic disease, malaria, extraintestinal amebiasis | Tumor necrosis factor | Inhibits the action of heme polymerase in malarial trophozoites, preventing the conversion of heme to hemazoin | 0.56954 | 0.7663 | 0.8063 |
|  | DB01611 | Hydroxy-chloroquine | Malaria, chronic discoid lupus, erythematosus, systemic lupus, acute rheumatoid arthritis, and chronic rheumatoid arthritis | Toll-like receptor 9,  Toll-like receptor 7,  DNA | Interferes with the malaria parasite’s ability to proteolyze haemoglobin, thus, inhibiting the normal growth and replication of the parasite  Interferes with the action of parasitic heme polymerase, allowing for the accumulation of the toxic beta-hematin  Inhibits adaptive immunity  Inhibits innate immunity | 0.39014 | 0.51466 | 0.58274 |
| Monoclonal antibody |  |  |  |  |  |  |  |  |
|  | DB08904 | Certolizumab pegol | Rheumatoid arthritis, psoriatic arthritis,  ankylosing spondylitis and Crohn’s disease | Tumor necrosis factor | Certolizumab acts as a neutralizer that targets the activation of TNF-alpha to inhibit the downstream inflammatory process. | 0.56393 | 0.56393 | 0.7877 |
|  | DB06674 | Golimumab | Rheumatoid arthritis,  psoriatic arthritis, ankylosing spondylitis, and ulcerative colitis | Tumor necrosis factor | Acts as an antibody that binds and inhibits TNF-alpha, thus preventing the ability of its target receptor. | 0.56393 | 0.7877 | 0.81003 |
|  | DB00065 | Infliximab | Crohn’s disease, ulcerative colitis, rheumatoid arthritis, ankylosing spondylitis, psoriatic arthritis, and plaque psoriasis | Tumor necrosis factor | Acts as an inhibitor that binds to TNF-alpha to prevent pro-inflammatory cascade signaling | 0.53534 | 0.7877 | 0.81003 |
|  | DB00051 | Adalimumab | Rheumatoid arthritis, juvenile idiopathic arthritis, psoriatic arthritis, ankylosing spondylitis, Crohn’s disease, ulcerative colitis, plaque psoriasis, non-infectious intermediate, posterior and panuveitis, hidradenitis suppurativa, and Pyoderma Gangrenosum | Tumor necrosis factor | Acts as an antibody that binds to TNF-alpha to inhibit its interaction with cell surface TNF receptors such as p55 and p75. | 0.53079 | 0.72775 | 0.75849 |
|  | DB01296 | Glucosamine | Osteoarthritis | Tumor necrosis factor,  Matrix metalloproteinase-9, Interferon-gamma  Chitosanase | Serves as a precursor | 0.52932 | 0.69089 | 0.74441 |
|  | DB00005 | Etanercept | Rheumatoid arthritis, plaque psoriasis, polyarticular idiopathic arthritis, psoriatic arthritis, and ankylosing spondylitis. | Tumor necrosis factor | Acts as an antibody that binds to TNF-alpha receptors, thus removing them from circulation and preventing their interaction with TNF-alpha | 0.52833 | 0.72005 | 0.75654 |
|  | DB05679 | Ustekinumab | Plaque psoriasis, psoriatic arthritis, Crohn’s disease, and ulcerative colitis. | Interleukin-12 subunit beta,  Interleukin-23 | Inhibits IL-12/23 signaling pathway leading to suppression of TH1 and TH17 cell lineage of cytokines and associated inflammatory pathways | 0.37554 | 0.4261 | 0.46755 |
|  | DB06168 | Canakinumab | Familial Cold Autoinflammatory Syndrome, Muckle-Wells Syndrome, juvenile idiopathic arthritis | Interleukin-1 beta | Binds to human IL-1β and neutralizes its inflammatory activity by blocking its interaction with IL-1 receptors | 0.36098 | 0.49231 | 0.55968 |
|  | DB09036 | Siltuximab | multicentric Castleman's disease, | Interleukin-6 | Binds with Interleukin-6 to prevent interacting with IL-6 receptors, thus inhibiting the proliferation of lymphocytes. | 0.36071 | 0.48012 | 0.5382 |
|  | DB06273 | Tocilizumab | Rheumatoid arthritis, giant cell arteritis, polyarticular juvenile idiopathic arthritis, systemic juvenile idiopathic arthritis, and cytokine release syndrome | Interleukin-6 receptor subunit alpha | Acts as an inhibitor and antibody that binds soluble and membrane-bound IL-6 receptors, preventing IL-6 mediated inflammation | 0.31537 | 0.4223 | 0.47212 |
|  | DB00108 | Natalizumab | multiple sclerosis. | Integrin alpha-4,  Low-affinity immunoglobulin gamma Fc region receptor III-B,  Intercellular adhesion molecule 1,  High-affinity immunoglobulin gamma Fc receptor I | inhibits the α4-mediated adhesion of leukocytes to their counter-receptor(s). | 0.31136 | 0.58395 | 0.62286 |
| Immunomodulators |  |  |  |  |  |  |  |  |
|  | DB08910 | Pomalidomide | Multiple myeloma | Tumor necrosis factor | Acts as an inhibitor that enhances T cell and natural killer cell-mediated immunity and inhibits the production of pro-inflammatory cytokines | 0.52095 | 0.69503 | 0.73025 |
|  | DB00668 | Epinephrine | Treatment of allergic reactions, idiopathic anaphylaxis, complete heart block, mucosal congestion, bronchial asthmatic paroxysms, syncope, Resuscitation in cardiac arrest following anesthetic accidents, open-angle glaucoma, relaxation of uterine musculature and inhibit uterine contractions | Tumor necrosis factor,  Alpha-1A adrenergic receptor,  Alpha-1B adrenergic receptor,  Beta-1 adrenergic receptor,  Beta-2 adrenergic receptor,  Alpha-2A adrenergic receptor,  Alpha-2B adrenergic receptor,  Alpha-1D adrenergic receptor | Acts as either antagonist or agonist. | 0.50493 | 0.65483 | 0.69113 |
|  | DB01041 | Thalidomide | Treatment of the cutaneous manifestations of moderate to severe erythema nodosum leprosum | Tumor necrosis factor,  Protein cereblon | Inhibits excessive tumor necrosis factor-alpha production and down-modulation of selected cell surface adhesion molecules | 0.40335 | 0.64995 | 0.68472 |
|  | DB00480 | Lenalidomide | Treatment of multiple myeloma, transfusion-dependent anemia | Tumor necrosis factor ligand superfamily member 11,  Protein cereblon,  Cadherin-5,  Prostaglandin G/H synthase 2, | Expresses immunomodulatory activity | 0.31162 | 0.41723 | 0.48212 |
|  | DB01250 | Olsalazine | Treatment of Inflammatory Bowel Disease and ulcerative colitis | Thiopurine S-methyltransferase,  Interferon-gamma | Involved in anti-inflammatory response | 0.3856 | 0.47881 | 0.53864 |
| Herbs and Natural products |  |  |  |  |  |  |  |  |
|  | DB01404 | Ginseng | Modern clinical research is inconclusive of its pharmacology. However, it is known to help the body resist stress. | Interleukin-6,  Prostaglandin G/H synthase 2,  Aryl hydrocarbon receptor | Acts as antagonist, inhibitor, or agonist | 0.3551 | 0.4769 | 0.52545 |
| Tetracycline |  |  |  |  |  |  |  |  |
|  | DB01017 | Minocycline | Treatment of inflammatory lesions of acne vulgaris, infections of susceptible microorganisms including rickettsiae, Mycoplasma pneumoniae, Chlamydia trachomatis, Chlamydophila psittaci, Chlamydia trachomatis, Ureaplasma urealyticum, Borrelia recurrentis, Haemophilus ducreyi, Yersinia pestis, Francisella tularensis, Vibrio cholerae, Campylobacter fetus, Brucella species, Bartonella bacilliformis, Klebsiella granulomatis, Escherichia coli, Enterobacter aerogenes, Shigella species, Acinetobacter species, Haemophilus influenzae, and Kelbsiella species | Interleukin-1 beta  Arachidonate 5-lipoxygenase,  Matrix metalloproteinase-9,  Vascular endothelial growth factor A,  Caspase-1,  Caspase-3,  Cytochrome c,  Mitogen-activated Protein Kinases,  Nitric oxide synthase inducible,  30S ribosomal protein S9,  30S ribosomal protein S4 | prevent aminoacyl-tRNA from binding to the 30S ribosome, inhibiting protein synthesis | 0.324 | 0.46679 | 0.54531 |
| Janus kinase (JAK) inhibitors |  |  |  |  |  |  |  |  |
|  | DB08877 | Ruxolitinib | Treatment of high-risk myelofibrosis, | Tyrosine-protein kinase JAK2, JAK1, JAK3 and Non-receptor tyrosine-protein kinase TYK2 | inhibits cell proliferation, induces apoptosis of malignant cells, and reduces pro-inflammatory cytokine plasma levels | 0.56393 | 0.44161 | 0.50625 |
|  | DB08895 | Tofacitinib | Treatment of rheumatoid arthritis, kidney transplant rejections, psoriasis, ulcerative colitis, and ankylosing spondylitis | Tyrosine-protein kinase JAK1, Tyrosine-protein kinase JAK2,  Tyrosine-protein kinase JAK3,  Non-receptor tyrosine-protein kinase TYK2 | prevent the body from responding to cytokine signals by regulating chemical signaling pathways | 0.31913 | 0.44454 | 0.50481 |
| Thrombolytic agents |  |  |  |  |  |  |  |  |
|  | DB00029 | Anistreplase | For lysis of acute pulmonary emboli, intracoronary emboli, and management of myocardial infarction | Plasminogen, Fibrinogen alpha chain,  Plasminogen activator inhibitor 1 | Anistreplase cleaves the Arg/Val bond in plasminogen to form plasmin | 0.31537 | 0.40083 | 0.44003 |
|  | DB00015 | Reteplase | For lysis of acute pulmonary emboli, intracoronary emboli, and management of myocardial infarction | Plasminogen, Fibrinogen alpha chain,  Plasminogen activator inhibitor 1 | Reteplase binds to fibrin-rich clots via the fibronectin finger-like domain and the Kringle 2 domain. The protease domain then cleaves the Arg/Val bond in plasminogen to form plasmin. | 0.31537 | 0.40083 | 0.44003 |
|  | DB00009 | Alteplase | For management of acute myocardial infarction, acute ischemic stroke, and lysis of acute pulmonary emboli | Plasminogen,  Fibrinogen alpha chain,  Urokinase plasminogen activator surface receptor,  Plasminogen activator inhibitor 1 | Binds to fibrin-rich clots and then cleaves the Arg/Val bond in plasminogen to form plasmin. | 0.31461 | 0.40083 | 0.44003 |
|  | DB00031 | Tenecteplase | Treatment of myocardial infarction and lysis of intracoronary emboli | Plasminogen, Fibrinogen alpha chain,  Urokinase plasminogen activator surface receptor,  Plasminogen activator inhibitor 1,  Plasminogen activator inhibitor 2,  Tetranectin,  Keratin, type II cytoskeletal 8,  Annexin A2,  Calreticulin,  Calnexin,  Prolow-density lipoprotein receptor-related protein 1 | Tenecteplase is a fibrin-specific tissue-plasminogen activator. Binds to fibrin-rich clots and then cleaves the Arg/Val bond in plasminogen to form plasmin. | 0.31119 | 0.41706 | 0.4763 |
