# Supplementary material for: Network-driven analysis of human–Plasmodium falciparum interactome: processes for malaria drug discovery and extracting in silico targets
Source: Malar J. 2021 Oct 26;20:421. doi: 10.1186/s12936-021-03955-0 (PMC8547565; doi:10.1186/s12936-021-03955-0)
Supplement: Supplementary file 10 — Additional file 10. Supplementary method. [file 12936_2021_3955_MOESM10_ESM.docx]

**Supplementary method**

# **Background**

Antimalarial resistance remains a major challenge to malaria control in Africa, a high transmission endemic region characterized by high disease reported cases and mortality. In this study, we aimed to identify potential protein targets to understand the mechanism of drug resistance and identify those molecular targets that can help design drugs that are more effective and more specific to the African populations through a comparative analysis of both host and *Plasmodium falciparum* genome.

# **Materials and Methods**

## *Scoring InterPro Datasets*

Similarity score $\left( X_{ij} \right)$ between a protein pair $\left( p_{i} \right)$ and $\left( p_{j} \right)$ with common signatures $\left( S_{k} \right)$is measured by the minimum number of the occurrence of these signatures. This is defined mathematically in **Equation 1** below,

$$X\equiv X_{ij}=\sum_{k=1}^{M} min\left\{ n_{ki}n_{kj} \right\}\left( 1 \right)$$

Where $n_{ki}$ and $n_{kj}$ represent the number of occurrences of signatures and $k$ is the number of proteins starting from 1 to the last$\left( M \right)$.

Due to the associated level of uncertainty in experimental datasets as a result of biases and noise, they naturally follow a normal distribution when compared to other distributions. This implies that, the datasets can be summarized by its mean $\left( \mu\right)$ and standard deviation $\left( \sigma\right)$. Also, this optimal distribution maximizes information entropy which is a measure of uncertainty within a dataset. It is defined as the average rate at which information is produced by a stochastic source of data. Therefore, as established by Mazandu and Mulder (1), the confidence level $\left( \delta\right)$ of the similarity score $\left( X \right)$ for scoring protein family and domain is defined as shown in **Equation 2**

$\delta\equiv\delta\left( X,\sigma,\alpha\right)=\emptyset\left( \frac{X^{\alpha}}{\sigma} \right)\left( 2 \right)$

where $\emptyset$ is the cumulative probability function of a normal distribution, $\alpha$ is the calibration control parameter which strengthens the impact of the confidence level. The scoring scheme rectifies the dataset to remove all outliers, thus maintaining data points that lie within a normal distance. After rectifying, the information entropy related to the dataset is computed using the binary entropy function shown in **Equation 3**

$$H_{2}\left( \delta\right)=-{\delta log}_{2}\left( \delta\right)-\left( 1-\delta\right){log}_{2}\left( 1-\delta\right)\left( 3 \right)$$

The scheme computes the functional relationship score between protein pairs sharing common signatures. The functional relationship score is defined as shown in **Equation 4**

$$\text{ Г(δ)=}1-H_{2}\left( \delta\right)\left( 4 \right)$$

The reliability or confidence score of the functional relationship between two proteins is defined as shown in **Equation 5**

$$R=\frac{\text{Г(δ)}}{\max_{s}\text{Г(s)}}\left( 5 \right)$$

## *Scoring Protein Sequence Similarity*

The scoring scheme presented uses the bit score between pair-wise homologous sequence alignments $\left( s_{1},s_{2} \right)$. The bit score, denoted as $\left( S\left( s_{1},s_{2} \right) \right),$provides a mean for defining homology between pairwise sequences by measuring the average information or features available per amino acid position in the aligned pair-wise sequence (1). Homology is simply defined as common evolutionary ancestry between genomic sequences (2). The bit score is obtained through pair-wise homologous sequence BLAST (3). BLAST sequence similarity estimates the bit score by identifying common features and estimating statistically significant similarity that reflects shared common ancestor (2). Also, the scheme presented uses the mutual information $I\left( s_{1},s_{2} \right)$ between pair-wise homologous sequence alignments $\left( s_{1},s_{2} \right)$. The mutual information is the underlying common substantial biological features contained in pair-wise homologous sequences (2). This information is based on the fundamental postulate about homologous sequences which is paraphrased as "the closer the similarity between a protein sequence pair, the closer in evolution" (4). This therefore implies that; the bit score is in direct relation with the mutual information between a protein sequence pair. Therefore, Mazandu et al. (1) established the relationship shown in **Equation 6** below.

$$S\left( s_{1},s_{2} \right)=\lambda I\left( s_{1},s_{2} \right)\left( 6 \right)$$

where $\lambda$is a constant defining the relationship between the pair of sequences.

The concept of homology is fundamental to in silico analysis of both DNA and protein sequences. However, the ability to establish homology when two sequences have more mutual information other than would be expected by chance is critical for the analysis (2). This is because homologous sequences do not always share significant sequence similarity (2). For example, some homologous protein alignments are not significant but these proteins are characterized as homologous based on statistical significant strong sequence similarity to intermediate sequence (2). For this reason, the scheme used measures the mutual biological evolution information (4) available per amino acid position to distinguish an alignment from chance using the relative entropy of target residue and background distributions shown as **Equation 7**

$$H\left( s_{1},s_{2} \right)=\sum_{i,j} q_{ij}s_{ij}=\sum_{i,j} q_{ij}{log}_{2}\left( \frac{q_{ij}}{q_{i}q_{j}} \right)\left( 7 \right)$$

where $q_{ij}$ is the target residue substitution frequency which is defined as the probability of finding a residue $i$ aligned with residue $j$ after a certain amount of evolution given that they both evolved from common ancestor who had residue $k$ at that position. $q_{i}$ is defined as the probability of occurrence of a residue $i$ in a set of sequences. $s_{ij}$ is the similarity score between residue i and j.

The reliability score for the pair-wise sequence similarity implemented in the algorithm used is defined as **Equation 8**

$$R\left( s_{1},s_{2} \right)=\frac{I\left( s_{1},s_{2} \right)}{max\{H\left( s_{1} \right),H\left( s_{2} \right)\}}\left( 8 \right)$$

where $H\left( s \right)$ is the relative entropy obtained after aligning protein sequence $s$ by itself.

## Computing topological centrality metrics

The degree metric which measures the ability of a node to interact or communicate directly with neighbouring nodes is represented as shown in **Equation 9**

$$degree\left( P \right)=\sum_{q\in N} \delta\left( p,q \right)\left( 9 \right)$$

where $p$ and $q$ are proteins and $N$ is the network.

$$\delta\left( p,q \right)=\{\begin{matrix} 1 if protein q is functionally linked to protein p \\ 0 otherwise \end{matrix}$$

The closeness measure which determines nodes that are relatively closer to all nodes in the network is represented as shown in **Equation 10**

$$C_{p}=\frac{\left| L_{c} \right|-1}{\left( n_{c}-1 \right)*S_{r}\left( p \right)}\left( 10 \right)$$

where $n_{c}$ is the number of nodes in the path of a node of interest and $\left| L_{c} \right|$ is the number of functional interactions connecting the nodes.

The betweenness centrality which is a measure of the influence that a node has over the flow of information within pair of nodes in the network is represented as shown in **Equation 11**

$$B\left( c \right)=\sum_{\left( a,b \right)\in N_{c}} \frac{\sigma_{ab}\left( c \right)}{\sigma_{ab}}\left( 11 \right)$$

where $\sigma_{ab}$ is the shortest paths between protein $a$ and $b$ passing through protein $c$. The shortest path between node pairs in a biological network is that path with the minimum number of edges.

# Kappa (equation 12), Jaccard (equation 13) and Best Match Average (BMA) (equation 14) similarity scores

$$SimKPS\left( p,q \right)=\frac{\sigma_{pq}-\alpha_{pq}}{1-\alpha_{pq}}\left( 12 \right)$$

where $\sigma_{pq}$ is defined as the observed as the observed frequency of co-occurrence between the profiles of protein p and q whereas $\alpha_{pq}$ is the likelihood of observing the profiles of protein p and q in the data under consideration (5).

$$SimGIC\left( p,q \right)=\frac{\sum_{t\in A_{p}\cap A_{q}} \text{IC}\left( t \right)}{\sum_{A_{p}\cup A_{q}} \text{IC}\left( t \right)}\text{ }\left( 13 \right)$$

$$\text{BMA}\left( p.q \right)=\frac{1}{2}\left( \frac{1}{n}\sum_{t\in A_{p}} \text{sim}\left( t,A_{q} \right)+\frac{1}{m}\sum_{t\in A_{q}} \text{sim}\left( t,A_{p} \right) \right) (14)$$

where $A_{p}$ and $A_{q}$ represent sets of the biological processes in which protein the proteins p and q, respectively, are involved, n and m are numbers of processes for p and p, and IC(t) is the information content of the biological process t, computed based on the structure of the gene ontology biological process. And $\text{sim}\left( t,A \right)=max\left\{ S\left( t,s \right)\text{ for }s\in A \right\}$ with S(t,s) the semantic similarity score between biological processes t and s, given by (equation 15):

$$S\left( t,s \right)=\frac{\text{ IC}\left( c \right)}{max\{IC\left( t \right),IC\left( s \right)\}} (15)$$

where c is the most informative common ancestor shared by t and s.

# Reference

1. Mazandu GK, Mulder NJ. Scoring protein relationships in functional interaction networks predicted from sequence data. PLoS One. 2011;6(4):e18607.

2. Stormo GD. An introduction to sequence similarity ("homology") searching. Curr Protoc Bioinformatics. 2009;Chapter 3:Unit 3 1 3 1 -7.

3. Mount DW. Using the Basic Local Alignment Search Tool (BLAST). CSH Protoc. 2007;2007:pdb top17.

4. Bastien O, Ortet P, Roy S, Marechal E. A configuration space of homologous proteins conserving mutual information and allowing a phylogeny inference based on pair-wise Z-score probabilities. BMC Bioinformatics. 2005;6:49.

5. Mazandu GK, Chimusa ER, Mulder NJJBB. Expressions of different semantic similarity measures in the context of biomedical sciences and WordNet. 2016;18(5):886-901.
